# Physical activity and the risk of cataract and age-related macular degeneration: a systematic review and meta-analysis of cohort studies

**DOI:** 10.1186/s12886-026-04721-z

**Published:** 2026-03-11

**Authors:** Dagfinn Aune, Ahmad Jayedi, Asma Kazemi, Sepideh Soltani, Fatemeh Rezaei, Michael F. Leitzmann

**Affiliations:** 1 Department of Epidemiology and Biostatistics, School of Public Health, Imperial College London, St Mary’s Campus, Norfolk Place, London, W2 1PG UK; 2 Department of Research, Cancer Registry of Norway, Norwegian Institute of Public Health, Oslo, Norway; 3 Department of Nutrition, Oslo New University College, Oslo, Norway; 4 Nutrition Research Center, School of Nutrition and Food Sciences, Shiraz University of Medical Sciences, Shiraz, Iran; 5 Cardiovascular Research Center, Shahid Sadoughi University of Medical Sciences, Yazd, Iran; 6 Research Center for Social Determinants of Health, Jahrom University of Medical Sciences, Jahrom, Iran; 7 Institute for Epidemiology and Preventive Medicine, University of Regensburg, Regensburg, Germany

**Keywords:** Physical activity, Cataract, Age-related macular degeneration, Cohort, Meta-analysis

## Abstract

**Background:**

Physical activity has been associated with a lower risk of cataract and age-related macular degeneration in some studies; however, the available evidence has not been fully consistent. We conducted a meta-analysis of cohort studies to clarify the association between physical activity and cataract and age-related macular degeneration.

**Methods:**

The PubMed and Embase databases were searched for relevant prospective studies up to September 18, 2025. Random effects models were used to calculate summary relative risks (RRs) and 95% confidence intervals (CIs) for the association between physical activity and the risk of cataract and age-related macular degeneration. World Cancer Research Fund (WCRF) criteria was used to evaluate the strength of evidence.

**Results:**

A total of 10 cohort studies (8 publications) with 163,065 cases and 1,914,137 participants were included in the analysis of physical activity and cataract and 14 cohort studies (9 publications) with 17,653 cases and 566,895 participants were included in the analysis of age-related macular degeneration. The summary RR for high vs. low physical activity and cataract was 0.90 (95% CI: 0.86–0.94, I^2^ = 74%, *n* = 10) and for age-related macular degeneration was 0.92 (95% CI: 0.84–1.01, I^2^ = 60%, *n* = 14). The summary RR per 20 MET-hours/week increment in leisure-time physical activity was 0.91 (0.84–0.99, I^2^ = 66%, *n* = 3) for cataract and 0.92 (0.74–1.13, I^2^ = 48%, *n* = 3) for age-related macular degeneration, and there was no indication of nonlinear dose-response relationships (p_nonlinearity_=0.32 and p_nonlinearity_=0.34, respectively). There was no indication of publication bias. The evidence (judging the likelihood of causality) using WCRF criteria was judged as probable for cataract and limited-no conclusion for age-related macular degeneration.

**Conclusion:**

This meta-analysis provides further support for an inverse association between physical activity and risk of cataract, but an association with age-related macular degeneration was less evident. Any further studies should clarify the dose-response relationship and associations between different domains of physical activity in relation to these outcomes. These findings support public health recommendations to the general population to be physically active.

**Supplementary Information:**

The online version contains supplementary material available at 10.1186/s12886-026-04721-z.

## Introduction

Cataract is the leading cause of blindness and impaired vision globally and accounts for 50% of all global blindness [1]. Age-related macular degeneration is the third leading cause of blindness globally [2]. While cataracts result from lens opacification and are typically reversible through surgery, age-related macular degeneration involves degenerative changes of the macula, leading to irreversible central vision loss. Globally 82 million people had cataracts in 2021, a substantial increase from 32.8 million in 1990 [3], and an estimated 196 million people had age-related macular degeneration in 2020 and this has been projected to increase to 288 million by 2040 [2]. Major risk factors for cataract include diabetes [4], higher body mass index (BMI, kg/m^2^) [4], hypertension [5], smoking [4], ultraviolet light [6], and steroid use [7], while some of the established risk factors for age-related macular degeneration include family history [8], cigarette smoking [9, 10], higher BMI [8], diabetes [10] and hypertension [10].

Given that metabolic risk factors including overweight and obesity, diabetes and hypertension appear to be associated with increased risk of cataract and age-related macular degeneration [4, 8], it could be hypothesized that physical activity may reduce risk, as physical activity has established benefits with regard to weight control [11], reductions in diabetes risk [12], and improvements in blood pressure [13]. However, studies on physical activity and cataract risk have shown somewhat mixed results [4, 14–19]. In the Beaver Dam Eye study [14], Women’s Health Initiative [15] and the EPIC-Oxford study [16], no clear associations were observed between physical activity and risk of cataract. In contrast, inverse associations were observed between higher physical activity and cataract in the National Runners’ Health Study [20], National Runners’ Health Study II and the National Walkers’ Health Study [17], the Swedish Mammography Cohort and Cohort of Swedish Men [18], Million Women Study [4], and the UK Biobank [19]. It is possible that an association was missed in some studies [14–16] because of limited statistical power, as the observed associations have generally been weak to moderate and sample sizes in these studies were moderate. Studies on physical activity and age-related macular degeneration have also shown mixed results [21–29], with four studies [22, 23, 27, 29] showing significant inverse associations, nine studies (5 publications) [21, 24, 25, 27, 28] reporting no statistically significant associations and one study reporting a positive association [26]. Given the inconsistency of the results between studies, we conducted a systematic review and meta-analysis of published studies to clarify the association between physical activity and risk of cataract and age-related macular degeneration.

## Methods

This review was reported in accordance with the PRISMA (Preferred Reporting Items for Systematic reviews and Meta-Analyses) statement [30].

### Search strategy

PubMed and Embase databases were searched for relevant studies up to September 18, 2025 on physical activity and the risk of cataract and age-related macular degeneration. The search strategy is shown in the Supplementary Text. We also hand searched the reference lists of the included studies, as well as previously published meta-analyses, for any further studies.

### Study selection and inclusion criteria

The screening was done in two phases, with titles and abstracts screened initially, and then full texts of potentially relevant articles were obtained for a final decision about inclusion or exclusion. We included retrospective and prospective cohort studies, nested case-control studies within cohort studies and case-cohort studies that reported adjusted relative risk (RR) estimates (risk ratios, hazard ratios, incidence rate ratios, odds ratios) for the association between physical activity and risk of cataract or age-related macular degeneration. When multiple publications were available from the same study, we used the publication with the largest number of cases or that provided the most detailed information. If the overlapping publications reported on different domains of physical activity or subtypes of the outcomes, both were included in the respective subgroup analyses. Studies with other study designs (e.g., retrospective case-control or cross-sectional studies), patient-based studies, studies reporting unadjusted risk estimates, and studies without data on physical activity and risk of cataract or age-related macular degeneration were excluded, and a list of the excluded studies and exclusion reasons is provided in Supplementary Table 1. DA conducted the literature screening using Reference Manager version 11.

### Data extraction

The following information was extracted from each study: name of the first author, publication year, geographic location, name of the study, recruitment and follow-up period, sample size, age, sex, number of cases, exposure (physical activity measure) and subgroup, frequency or level of activity, relative risks (95% confidence intervals), and confounders adjusted for in the analysis. The extracted data are shown in Supplementary Tables 2 and 3. DA extracted the data, and AJ checked the data extractions for accuracy.

### Quality assessment of included studies

A modified version of the Newcastle-Ottawa scale for cohort studies was used to assess the quality of the included observational studies [31]. The modified scale provided a total score ranging from 0 to 8 points, with scores of 0–3, > 3–6 and > 6–8 indicating low, medium and high study quality, respectively. The modifications included: [1] removing the item on representativeness, as it was not considered relevant for study quality [2], assigning 0.25 points per confounder adjusted for (up to a maximum of 2 points), instead of 1 point for each of two confounders, to avoid granting the maximum score to studies that adjusted only for age and sex, and [3] awarding one point for outcome assessment based on registry linkage. The study quality assessments are displayed in Supplementary Tables 4 and 5. DA and AJ did the study quality assessment in duplicate.

### Assessment of the strength of evidence

We used criteria developed by the World Cancer Research Fund to evaluate the likelihood of causality [32]. These criteria are outlined in detail in Supplementary Tables 6 and judges the probability that an observed association is causal based on several factors, including the size and precision of the summary estimates, the number of published studies, heterogeneity, dose-response relationship, evidence from different study types, study quality, experimental evidence and biological plausibility. The following evidence grades are available: (1) substantial effect on risk unlikely, (2) limited-no conclusion, (3) limited-suggestive, (4) probable and (5) convincing evidence of a causal relationship [32].

### Statistical methods

The random effects model by DerSimonian and Laird, which take into account heterogeneity within and between studies, was used to calculate summary RRs and 95% confidence intervals (CIs) for the association between physical activity and cataract and age-related macular degeneration [33]. Because of differences in the way the physical activity data were reported between studies, and because few studies could therefore be included in the dose-response analysis, our primary analyses were based on the highest vs. lowest level of activity (as defined and reported in each individual study; See Supplementary Tables 2 and 3 for detailed descriptions of cut-off points across studies). For the primary analysis we used estimates unadjusted for BMI, when available, as adiposity could be a mediating factor between physical activity and cataract and age-related macular degeneration. The method of Greenland and Longnecker [34] was used for the linear dose-response analysis to estimate study-specific slopes (linear trends) and 95% CIs from the natural logarithm of the RRs across categories of physical activity. When ranges of physical activity were reported, the width of the adjacent category was used to estimate a lower or upper cut-off point for open-ended categories and when the upper boundaries were extreme. Nonlinear dose-response analyses were conducted to examine the shape of the dose-response relationship. The nonlinear dose-response analyses were conducted using restricted cubic splines, with three knots at 10%, 50%, and 90% centiles of the distribution, which were combined using multivariable meta-analysis [35]. For three studies [17, 20, 22], we used a plotdigitizer (www.plotdigitizer.com) to read data on running distance (*n* = 2) and Metabolic Equivalent Task (MET)-hours/week of walking and running (*n* = 1) in relation to cataract risk. For one of these studies [17] that reported results for MET-hours/week, we considered walking and running to approximate total leisure-time physical activity for inclusion in the dose-response analysis. Dose-response analyses were only possible for a small subset of the studies because of the way the data were reported (e.g. often dichotomous categories or no quantification of the physical activity level). Because definitions of physical activity varied across studies, we included all measures reflecting total or domain-specific activity to capture overall movement behaviour in the primary analysis. We conducted additional subgroup analyses by physical activity type and also reviewed results for physical types where there was only one study to ensure consistency.

Heterogeneity between studies was evaluated with Q- and I-squared (I^2^) statistics [36]. Subgroup and meta-regression analyses were conducted to investigate possible sources of heterogeneity, including sex, duration of follow-up, geographic location, number of cases, outcome type (for age-related macular degeneration: total, early, intermediate, late, dry, wet), study quality, and adjustment for confounding factors. Sensitivity analyses were conducted to investigate the robustness of the findings by excluding one study at a time from the meta-analysis to test whether the results were driven by a single large study or by outliers. E-values were calculated to estimate the minimum strength of association that an unmeasured or uncontrolled confounder would need to have with both the exposure and the outcome to fully explain away the observed associations [37]. Publication bias was assessed with Egger’s test [38], and by inspection of funnel plots [39]. We also conducted a further sensitivity analysis, converting the odds ratios to RRs for one study on cataract [15] and three studies on age-related macular degeneration [21, 23, 25] that reported sufficient information to make such a conversion (one additional study on age-related macular degeneration [28] did not provide sufficient information for such a re-calculation). This re-calculation was done using the following formula: RR = OR / 1 - risk_0_ + (risk_0_ x OR), where risk_0_ is the baseline risk in the unexposed group, and OR is the odds ratio. All statistical analyses were conducted using STATA version 17.0 (StataCorp, College Station, TX, USA).

## Results

**Fig. 1:**
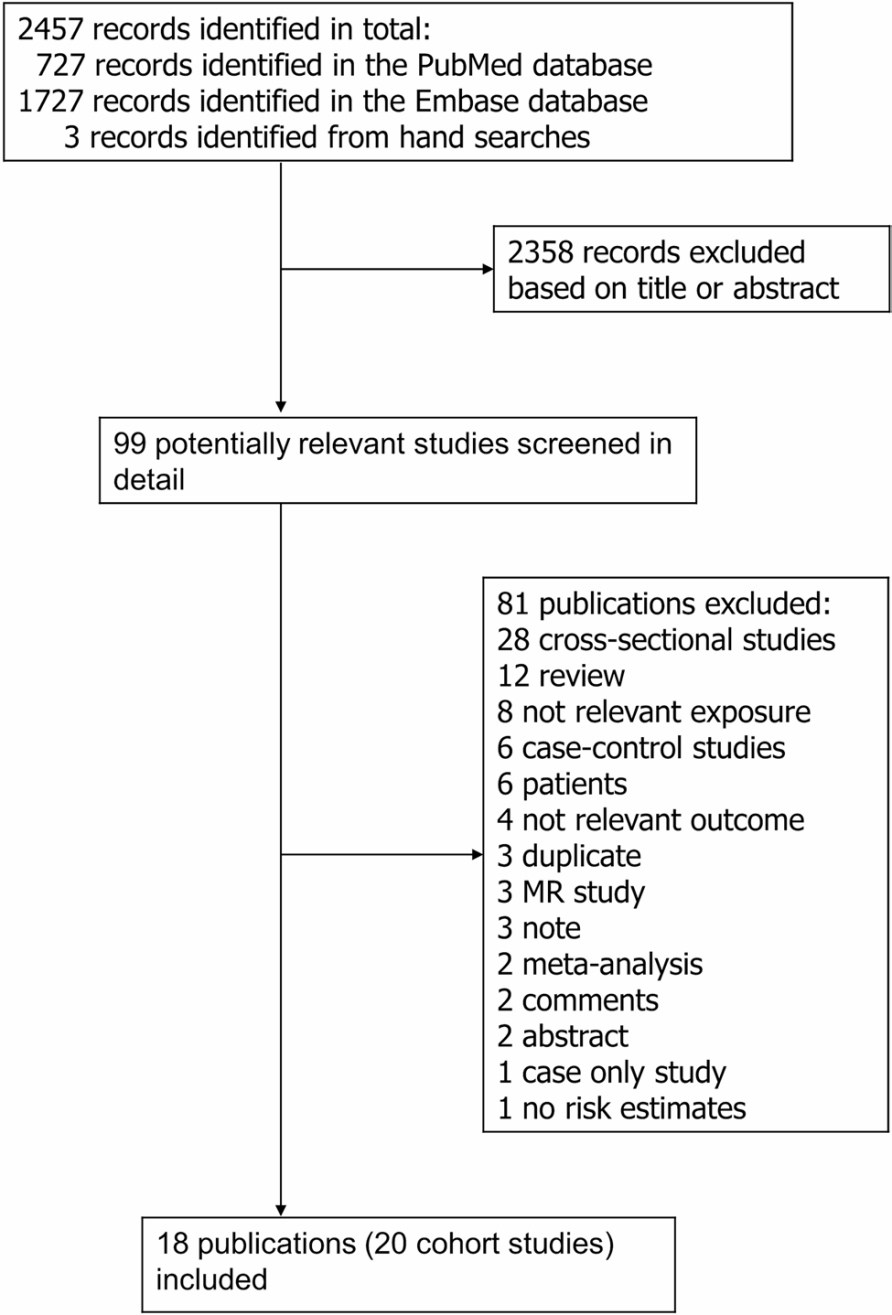
Flow-chart of study selection

Out of a total of 2457 records screened, 99 articles were examined in more detail, and 18 publications [4, 14–29, 40] with data from 20 cohort studies were included in the meta-analysis (Fig. 1). Two publications included two studies each [17, 18], and one publication included six studies that were used [27]. For one study (Blue Mountains Eye Study), we used one publication with sufficient information for the dose-response analysis [40], while a more recent study was used for the high vs. low analysis, as it reported both on early and late age-related macular degeneration [25]. The characteristics extracted from the included studies are presented in Supplementary Tables 2 and 3. The duration of follow-up ranged from 7 to 12.1 years for studies on cataract and from 3 to 15.5 years for studies on age-related macular degeneration. The age of the participants ranged from 40 to 86 years for the studies on cataract and from 20 to 96 years for the studies on age-related macular degeneration. Among the ten studies on cataract, there were three studies including only women, one study including only men, six studies including both men and women; five studies were from the USA, and five were from Europe (three were from the United Kingdom and two from Sweden). All fourteen studies on age-related macular degeneration included both men and women, and eight were from Europe, three were from the US, two were from Australia, and one was from Asia (South Korea).

### Study quality assessment

The studies included on physical activity and cataract had a mean (median) quality score of 5.7 (5.9) points out of a maximum total of 8 points, ranging from 3.25 to 8 (Supplementary Table 4). This indicates a moderately high methodological quality of the studies. The main quality issues were a lack of information on whether the physical activity assessment had been validated, missing information on the proportion of participants lost to follow-up, and lack of description of whether participants with a prevalent diagnosis of the outcome had been excluded or not at baseline (Supplementary Table 4). The studies included on physical activity and age-related macular degeneration had a mean (median) quality score of 4.9 (4.5), suggesting moderate methodological quality of the studies (Supplementary Table 5). The main quality issues were lack of information on the validity of the physical activity assessment, lack of information on exclusion of prevalent cases at baseline, lack of information on the proportion of participants lost to follow-up, and suboptimal confounder adjustments (Supplementary Table 5).

### Physical activity and cataract

**Fig. 2:**
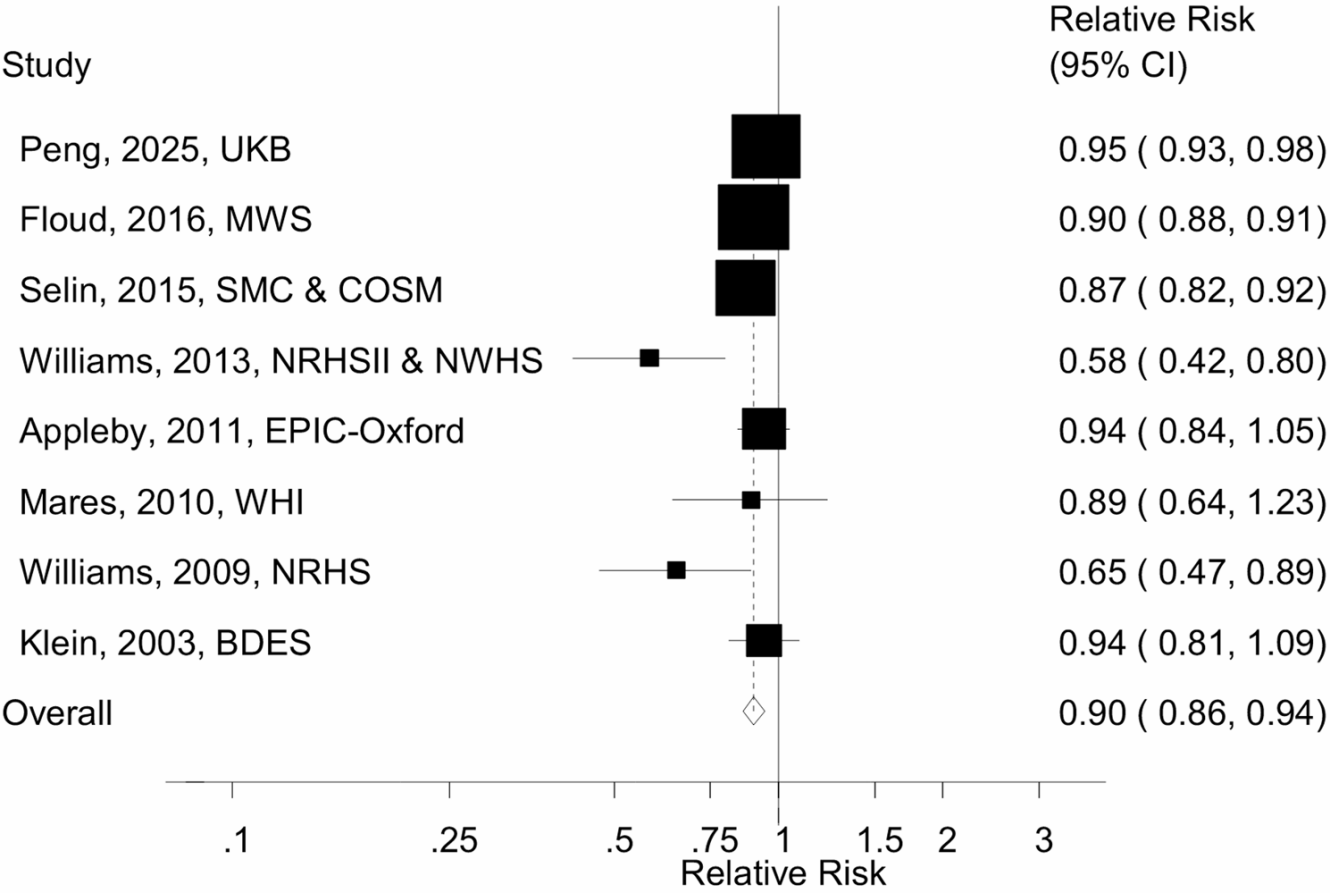
Physical activity and cataract

Ten cohort studies (8 publications, 8 risk estimates) [4, 14–20] with 163,065 cases and 1,914,137 participants were included in the analysis of physical activity and cataract risk. The summary RR for high vs. low physical activity was 0.90 (95% CI: 0.86–0.94, I^2^ = 74%) (Fig. 2). The summary RR ranged from 0.88 (0.83–0.93) when excluding the study by Peng et al. [19], to 0.91 (0.87–0.95) when excluding the study by Williams [17] (Supplementary Fig. 1). The heterogeneity was reduced to 19% when excluding two outlying studies [17, 19], and the summary RR remained similar (RR = 0.89, 0.87–0.92). There was no indication of publication bias with Egger’s test (*p* = 0.39) or by inspection of the funnel plot (Supplementary Fig. 2).

**Fig. 3:**
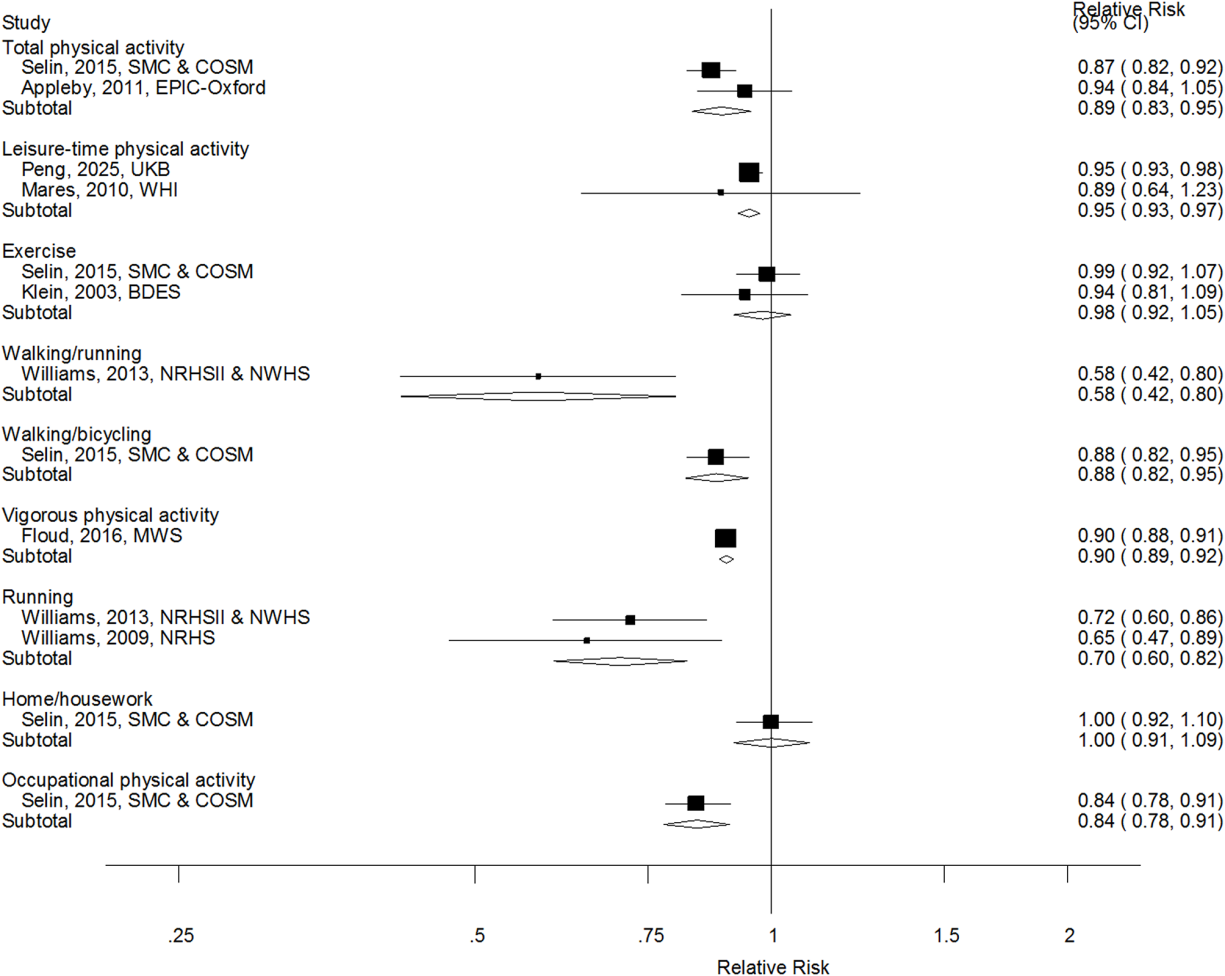
Physical activity subtypes and cataract

**Fig. 4:**
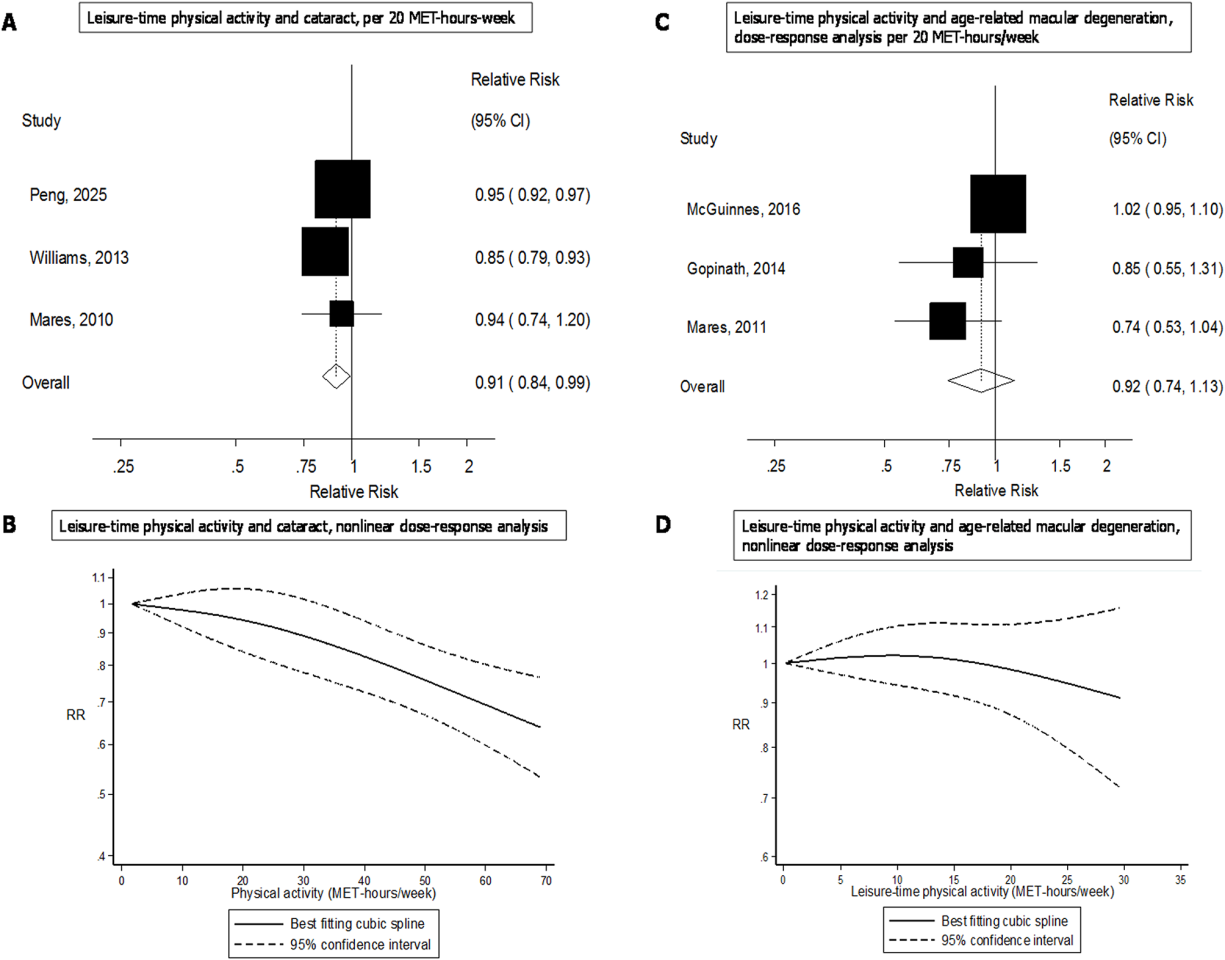
Leisure-time physical activity and cataract and age-related macular degeneration, linear and nonlinear dose-response analyses

When specific subtypes of physical activity were analysed, inverse associations were observed for total physical activity (summary RR = 0.89, 0.83–0.95, I^2^ = 32%, *n* = 2), leisure-time physical activity (summary RR = 0.95, 0.93–0.97, I^2^ = 0%, *n* = 2), running (summary RR = 0.70, 0.60–0.82, I^2^ = 0%, *n* = 2) and for some additional types of activity based on single studies (Fig. 3). Three studies were included in the dose-response analysis of leisure-time physical activity and cataract [15, 17, 19] and the summary RR per 20 MET-hours/week was 0.91 (0.84–0.99, I^2^ = 66%) (Fig. 4a), and there was evidence of a clear inverse dose-response relationship, but no indication of nonlinearity (p_nonlinearity_=0.32) (Fig. 4b, Supplementary Table 7).

### Physical activity and age-related macular degeneration

**Fig. 5:**
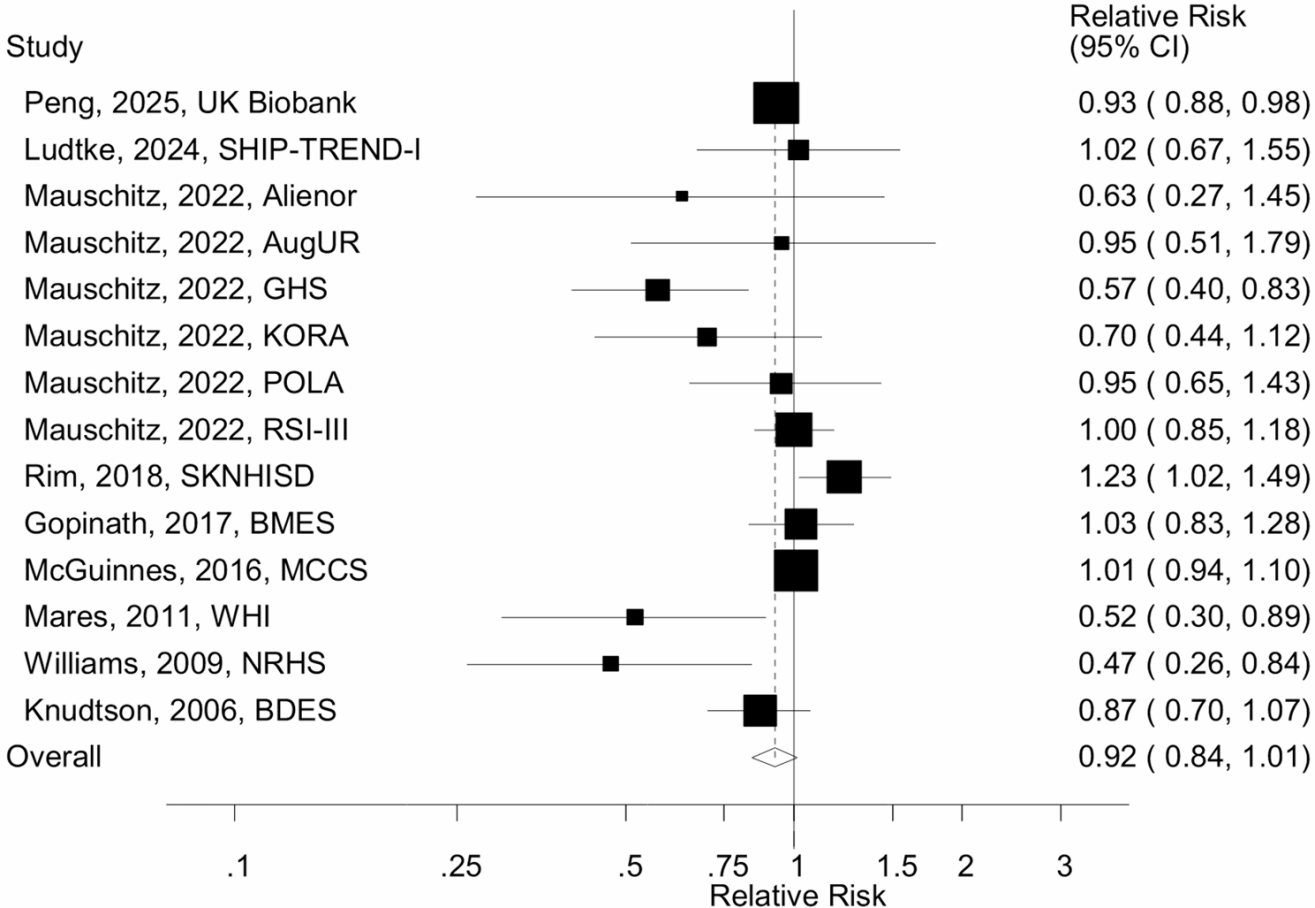
Physical activity and age-related macular degeneration

Fourteen cohort studies (9 publications) [21–29] with 17,653 cases and 566,895 participants were included in the analysis of physical activity and risk of age-related macular degeneration. The summary RR for high vs. low physical activity was 0.92 (95% CI: 0.84–1.01, I^2^ = 60%) (Fig. 5). The summary RR ranged from 0.89 (0.80-1.00) when the study by McGuinness et al. [24] was excluded to 0.95 (0.88–1.04) when the Gutenberg Health Study by Mauschitz et al. [27] was excluded (Supplementary Fig. 3). There was no evidence of publication bias with Egger’s test (*p* = 0.18) or by inspection of the funnel plot (Supplementary Fig. 4).

**Fig. 6:**
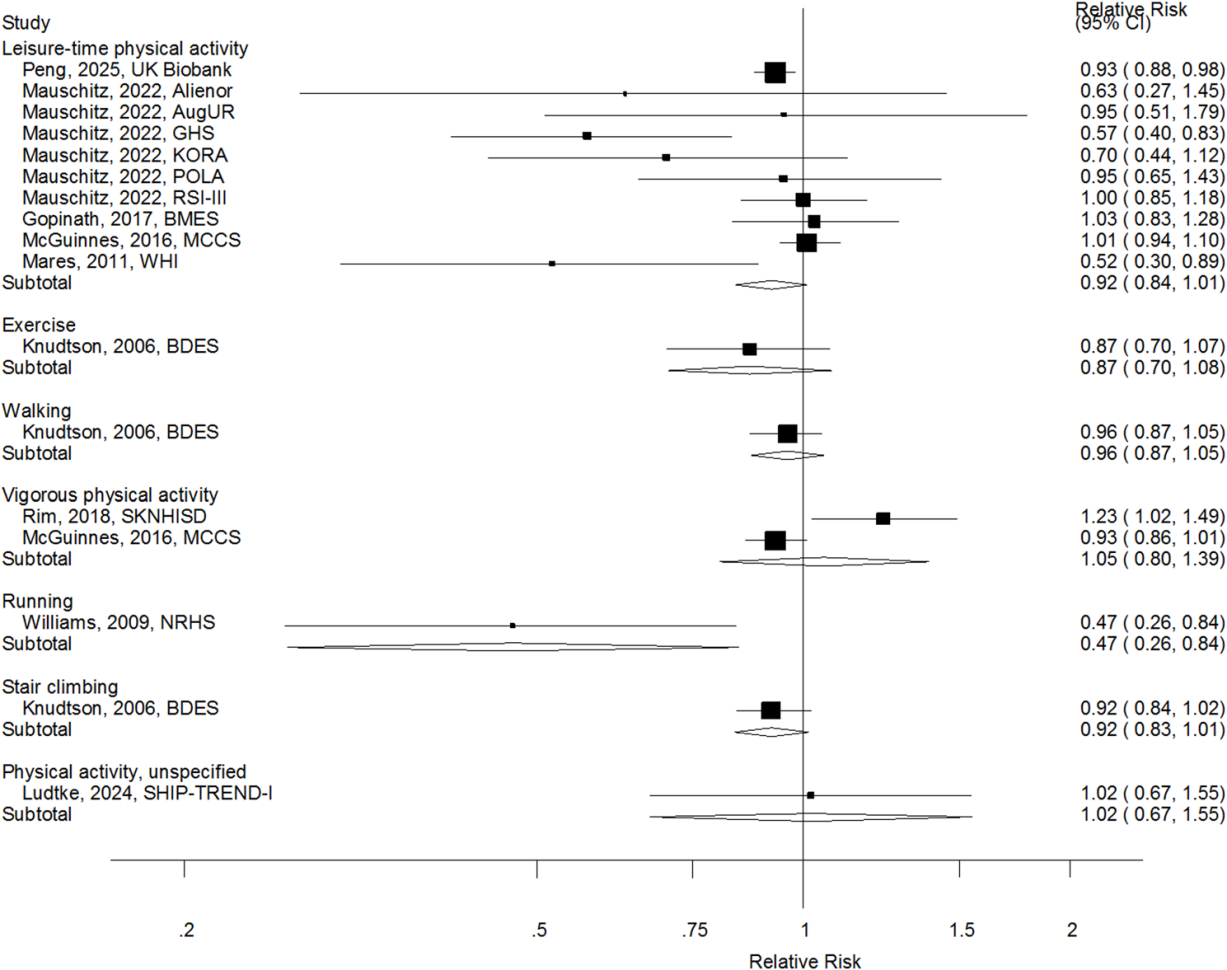
Physical activity subtypes and age-related macular degeneration

When specific subtypes of physical activity were investigated, the summary RR was 0.92 (0.84–1.01, I^2^ = 52%, *n* = 10) for leisure-time physical activity, and 1.05 (0.80–1.39, I^2^ = 86%, *n* = 2) for vigorous physical activity (Fig. 6).

Dose-response analyses were possible only for leisure-time physical activity, and three cohorts [23, 24, 40] were included in that analysis. The summary RR per 20 MET-hours/week increment in leisure-time physical activity was 0.92 (0.74–1.13, I^2^ = 48%) (Fig. 4c). There was no indication of a nonlinear dose-response relationship (p_nonlinearity_=0.34) (Fig. 4d, Supplementary Table 7).

### Subgroup analyses, sensitivity analyses and E-values

The inverse association between physical activity and cataract persisted across most subgroups, and there was little indication of heterogeneity between subgroups. Only in the subgroups stratified by duration of follow-up and adjustment for fruits and vegetables was there significant heterogeneity between subgroups, with stronger associations in studies with shorter vs. longer follow-up (p_heterogeneity_=0.04) and among studies with vs. without adjustment for fruit and vegetables (p_heterogeneity_=0.02) (Supplementary Table 8). The null association between physical activity and age-related macular degeneration persisted across most subgroups, and there was no between-subgroup heterogeneity observed (Supplementary Table 8).

A further sensitivity analysis where estimates from studies reporting odds ratios were re-calculated to relative risks was conducted, but this did not substantially alter the summary estimates, with the summary estimate for high vs. low physical activity and cataract remaining identical (0.90, 0.86–0.94) and for age-related macular degeneration being only slightly modified (from 0.92, 0.84–1.01 to 0.93, 0.85–1.01).

The E-value for the association between physical activity and cataract was 1.46 (lower CI: 1.31).

### Evidence grading

Based on World Cancer Research Fund grading criteria (Supplementary Table 6), we graded the evidence that physical activity reduces the risk of cataract as probably causal, while the evidence for an association between physical activity and age-related macular degeneration was graded as limited, and no conclusion could be drawn (Supplementary Table 9). A justification for this grading is shown in Supplementary Table 10.

## Discussion

In this meta-analysis of cohort studies, we found a 10% lower risk of developing cataract among persons with high vs. low levels of physical activity, but an association between higher physical activity and age-related macular degeneration was less evident. There was evidence of a strong inverse dose-response relationship between higher leisure-time physical activity and cataract risk, and there was a 25% reduction in risk at 50 MET-hours/week. These results persisted in sensitivity analyses excluding one study at a time, and there was no indication of publication bias.

Our analysis is consistent with a previous meta-analysis published in 2017 on cataract, but included a much larger number of cases (163,065 vs. 19,173) and participants (1,914,137 vs. 171,620) [41]. Our analysis of age-related macular degeneration differs somewhat from a previous meta-analysis, also published in 2017, that found an inverse association between physical activity and age-related macular degeneration [42]. In the current analysis the association was not statistically significant, although we cannot exclude a small reduction in risk of a comparable size to what was previously observed. This discrepancy may partly be due to the inclusion of additional, more recent cohorts reporting positive [26] or null [25, 27, 28] associations.

Our meta-analysis has both limitations and strengths. Some heterogeneity between studies is expected as different studies had differences in the level and intensity of physical activity, detail of assessment of physical activity, background rates of cataract or age-related macular degeneration, age of the populations, duration of follow-up, sample size and number of cases, adjustment for confounding factors, or potential over-adjustment for mediating factors, all factors which could potentially contribute to heterogeneity in the associations between physical activity and cataract or age-related macular degeneration between studies. However, it is difficult to assess the relative impact of each of these factors robustly without having access to the original data. We observed high heterogeneity in the association between physical activity and cataract risk. However, this was due to differences in the strength rather than the direction of the associations, as all studies reported RRs below 1.00. When excluding two outlying studies, the heterogeneity was substantially reduced, but results remained similar. We cannot exclude the possibility that residual confounding could explain or contribute to the inverse association observed between physical activity and cataract risk. However, most of the cohort studies adjusted for a range of important confounding factors, including BMI, smoking, alcohol, and hormone therapy, and the overall association persisted in subgroup analyses stratified by adjustment for confounders. The E-value for the association between physical activity and cataract was 1.46 (lower CI: 1.31), suggesting an unadjusted risk factor would need to have a moderate association with both physical activity and cataract risk to fully explain away the observed association. Only one study adjusted for sun exposure, but still reported a reduced risk of cataract with higher physical activity after such adjustment [19]. Given the weak to moderate association between sun exposure and cataract [43], it seems less likely that confounding from sun exposure could fully explain away the observed association. It is possible that certain factors that were adjusted for in some studies, including adiposity, hypertension and diabetes, may be on the causal pathway between physical activity and cataract, and thus represent overadjustments. Further studies should investigate whether these factors may mediate part of the observed associations. Differences in the detail of the physical activity assessment may also have contributed to heterogeneity in the observed results; however, detailed information on physical activity was frequently not reported in the included studies, as physical activity was often one of several risk factors investigated. Given the inconsistencies in how the physical activity data were reported, we could only conduct dose-response analyses for a small subset of the included studies, and these analyses should be interpreted with caution. Further studies are needed to clarify the dose-response relationship between physical activity and cataract and age-related macular degeneration risk, and more standardized physical activity quantification (e.g. MET-hours/week) across studies would be useful. Additional research is also needed to explore the associations with specific domains of physical activity, as the number of studies was limited. Measurement errors in both the exposure and outcome may have affected the results, however, given the cohort design of the included studies, any bias would most likely have attenuated the associations toward the null. Since this was a meta-analysis of published studies, we cannot exclude the possibility that publication bias may have affected the results. However, we found no indication of publication bias based on Egger’s test or visual inspection of the funnel plots. In addition, statistical power was considerably lower for age-related macular degeneration than in the analysis of cataract because the studies were of small to moderate size and the number of cases was considerably smaller than in the analysis of cataract (17,653 cases vs. 163,065 cases). Given the limited data on subtypes of age-related macular degeneration, these findings should be considered preliminary and further studies are needed before a firm conclusion can be made.

Strengths of the meta-analysis include a comprehensive literature search, detailed subgroup and sensitivity analyses, and the robustness of the main findings in sensitivity analyses. Also, the temporal design of the included cohort studies supports the plausibility that higher physical activity precedes a lower risk of lens opacification. Since most of the available evidence was from Europe and the U.S., further studies are needed from other regions.

Several biological mechanisms could explain the observed association between physical activity and cataract risk. Physical activity is associated with improved weight control and reduced risk of overweight/obesity and weight gain [11], and adiposity is a risk factor for cataract development [4, 44] and possibly age-related macular degeneration [44]. In three studies, further adjustment for baseline BMI had only a small attenuating effect on the associations between physical activity and risk of cataract (e.g. 7–20% reductions in the strength of the associations) [17, 20], but slightly strengthened associations with age-related macular degeneration [22]. There is also strong evidence for a benefit of physical activity in the prevention of type 2 diabetes [12], and a history of diabetes is associated with a roughly 3-fold increase in risk of cataract [4] and also elevated risk of age-related macular degeneration [45]. Physical activity has established benefits in relation to blood pressure lowering [13], and hypertension is an established risk factor for both cataract [5] and age-related macular degeneration [10]. Metabolic risk factors such as elevated blood glucose, insulin resistance and dyslipidemia may contribute to lens opacities through oxidative damage and formation of advanced glycation end products, while physical activity may reduce ocular oxidative stress and inflammation [46]. One cross-sectional study reported that physical activity was associated with reduced risk of precursors of age-related macular degeneration [47].

These findings have important public health implications as the prevalence of cataract globally has increased over the last few decades in parallel with the obesity epidemic. Increasing physical activity levels in the general population could therefore be an important strategy for the primary prevention of cataract, and such a strategy could also have benefits for a large number of other chronic diseases [12, 48–52].

In conclusion, we found a 10% reduction in risk of cataract with high vs. low physical activity and a 25% reduction in risk at 50 MET-hours/week, but an association with age-related macular degeneration was less evident. Further studies are needed to clarify the dose-response relationship between physical activity and different domains of physical activity and the risk of cataract and age-related macular degeneration.

## Supplementary Information

Below is the link to the electronic supplementary material.

Supplementary Material 1

## Data Availability

The data used are described in the article. Datasets and code are available from the corresponding author upon request.

## Abbreviations

BMI: Body mass index
CI: Confidence intervals
EPIC: European Prospective Investigation into Cancer and Nutrition
MET: Metabolic equivalent task
PRISMA: Preferred Reporting Items for Systematic reviews and Meta-Analyses
RR: Relative risk
WCRF: World Cancer Research Fund
