# Supplementary material for: Physical activity and the risk of cataract and age-related macular degeneration: a systematic review and meta-analysis of cohort studies
Source: BMC Ophthalmol. 2026 Mar 11;26:194. doi: 10.1186/s12886-026-04721-z (PMC13094163; doi:10.1186/s12886-026-04721-z)
Supplement: Supplementary file 1 — Supplementary Material 1 [file 12886_2026_4721_MOESM1_ESM.docx]

Supplement for:

Aune D, Jayedi A, Kazemi A, Soltani S, Rezaei F, Leitzmann MF. Physical activity and the risk of cataract and age-related macular degeneration: a systematic review and meta-analysis of cohort studies.

Supplementary Text. Search strategy in PubMed and Embase

PubMed search terms:

1) ("physical activity[MeSH Terms] OR exercise[MeSH Terms] OR sports[MeSH Terms] OR walking[MeSH Terms] OR biking[MeSH Terms] OR running[MeSH Terms] OR fitness[MeSH Terms] OR exercise test[MeSH Terms] OR inactivity[MeSH Terms] OR sedentary[MeSH Terms])

2) ) ("physical activity[Title/Abstract] OR exercise[Title/Abstract] OR sports[Title/Abstract] OR walking[Title/Abstract] OR biking[Title/Abstract] OR running[Title/Abstract] OR fitness[Title/Abstract] OR exercise test[Title/Abstract] OR inactivity[Title/Abstract] OR sedentary[Title/Abstract])

3) (cataract[MeSH Terms] OR lens opacity[MeSH Terms] OR lens opacities[MeSH Terms] OR macular degeneration[MeSH Terms])

4) (cataract[Title/Abstract] OR lens opacity[Title/Abstract] OR lens opacities[Title/Abstract] OR macular degeneration[Title/Abstract])

5) 1 OR 2

6) 3 OR 4

7) 5 AND 6

Embase search terms:

1) physical activity/ or cycling/ or jogging/ or running/ or walking/ or bicycle/ or sport/ or exercise/ or fitness/ or sedentary lifestyle/

2) (physical activity or cycling or jogging or running or walking or bicycle or sport or exercise or fitness or sedentary lifestyle).ab,ti.

3) cataract/

4) exp cataract/co, ep, et, pc [Complication, Epidemiology, Etiology, Prevention]

5) macular degeneration/

6) exp macular degeneration/co, ep, et, pc [Complication, Epidemiology, Etiology, Prevention]

7) (cataract or lens opacity or macular degeneration).ab,ti.

8) 1 OR 2

9) 3 OR 4

10) 8 AND 9

Supplementary Table 1. List of excluded studies and exclusion reasons

| Exclusion reason | Reference number |
| --- | --- |
| Abstract | (1;2) |
| Case-control study | (3-8) |
| Case only study | (9) |
| Comment | (10;11) |
| Cross-sectional study | (12-39) |
| Duplicate | (40-42) |
| Meta-analysis | (43;44) |
| Mendelian Randomization study | (45-47) |
| No risk estimates | (48) |
| Not relevant exposure | (49-56) |
| Not relevant outcome | (57-60) |
| Note | (61-63) |
| Patients | (64-69) |
| Review | (70-81) |

Reference List

1. Finger RP, McGuinness M, Hodge A, Giles G, Robman L, Guymer RH. Physical activity and age-related macular degeneration. Investigative Ophthalmology and Visual Science Conference: 2015;01.

2. Wang W, Shang X, Zhang L, Li L, He M. Risk factors for early-onset cataracts treated surgically in Australian adults. Investigative Ophthalmology and Visual Science Conference: 2018;01.

3. Paunksnis A, Kusleika S, Kusleikaite M. The relationship of the intensity of lens opacity with physical activity. Medicina (Kaunas) 2006;42:738-43.

4. Ristau T, Ersoy L, Hahn M et al. Nongenetic risk factors for neovascular age-related macular degeneration. Invest Ophthalmol Vis Sci 2014;55:5228-32.

5. Nunes S, Alves D, Barreto P et al. Adherence to a Mediterranean diet and its association with age-related macular degeneration. The Coimbra Eye Study-Report 4. Nutrition 2018;51-52:6-12.

6. Zult T, Smith L, Stringer C, Pardhan S. Levels of self-reported and objective physical activity in individuals with age-related macular degeneration. BMC Public Health 2020;20:1144.

7. Rim PHH, de Vasconcellos JPC, de Melo MB et al. Correlation between genetic and environmental risk factors for age-related macular degeneration in Brazilian patients. PLoS One 2022;17:e0268795.

8. Dev MK, Black AA, Cuda D, Wood JM. Low Light Exposure and Physical Activity in Older Adults With and Without Age-Related Macular Degeneration. Translational Vision Science and Technology 11(3) (no pagination), 2022;21.

9. Ravasco P, Ferreira C, Almeida AI, Ornelas M, Camilo M, Monteiro GM. Physical activity, nutritional status and body composition: Risk factors for cataract? Clinical Nutrition, Supplement Conference: 34th European Society for Clinical Nutrition and Metabolism, ESPEN Congress Barcelona Spain 7(1) (pp 97), 2012;September.

10. McGuinness MB, Simpson JA, Finger RP. Analysis of the Association Between Physical Activity and Age-Related Macular Degeneration. JAMA Ophthalmol 2018;136:139-40.

11. Liu L, Majithia S, Tham YC. Physical Activity and Age-related Macular Degeneration: A Systematic Literature Review and Meta-analysis. Am J Ophthalmol 2018;185:123.

12. Carresi C, Cruciani F, Paolucci F et al. Montelparo study: risk factors for age-related macular degeneration in a little rural community in Italy. La Clinica terapeutica 160(3) (pp e43-51), 2009;2009.

13. Moon BG, Joe SG, Hwang JU, Kim HK, Choe J, Yoon YH. Prevalence and risk factors of early-stage age-related macular degeneration in patients examined at a health promotion center in Korea. J Korean Med Sci 2012;27:537-41.

14. Munch IC, Linneberg A, Larsen M. Precursors of age-related macular degeneration: associations with physical activity, obesity, and serum lipids in the inter99 eye study. Invest Ophthalmol Vis Sci 2013;54:3932-40.

15. Erke MG, Bertelsen G, Peto T, Sjølie AK, Lindekleiv H, Njølstad I. Cardiovascular risk factors associated with age-related macular degeneration: the Tromsø Study. Acta Ophthalmol 2014;92:662-9.

16. Moshfeghi AA, Vaziri K, Moshfeghi DM. Patient behaviors and risk factors for age-related macular degeneration: Findings from the behavioral risk factor surveillance system. Investigative Ophthalmology and Visual Science Conference: 2014;01.

17. Khotcharrat R, Patikulsila D, Hanutsaha P et al. Epidemiology of Age-Related Macular Degeneration among the Elderly Population in Thailand. J Med Assoc Thai 2015;98:790-7.

18. Nguyen AM, Arora KS, Swenor BK, Friedman DS, Ramulu PY. Physical activity restriction in age-related eye disease: a cross-sectional study exploring fear of falling as a potential mediator. BMC Geriatr 2015;15:64.

19. Loprinzi PD, Swenor BK, Ramulu PY. Age-Related Macular Degeneration Is Associated with Less Physical Activity among US Adults: Cross-Sectional Study. PLoS One 2015;10:e0125394.

20. Marra KV, Kwiatkowski M, Yu G, Arroyo JG. Physical activity and age-related macular degeneration: Results from the 2005-2006 national health and nutrition examination surveys. Investigative Ophthalmology and Visual Science Conference: 2015;01.

21. Subhi Y, Sørensen TL. Physical activity patterns in patients with early and late age-related macular degeneration. Dan Med J 2016;63.

22. Nam SW, Lim DH, Cho KY, Kim HS, Kim K, Chung TY. Risk factors of presenile nuclear cataract in health screening study. BMC Ophthalmol 2018;18:263.

23. Raimundo M, Mira F, Cachulo MDL et al. Adherence to a Mediterranean diet, lifestyle and age-related macular degeneration: the Coimbra Eye Study - report 3. Acta Ophthalmol 2018;96:e926-e932.

24. Zhang K, Zhong Q, Chen S et al. An epidemiological investigation of age-related macular degeneration in aged population in China: the Hainan study. International Ophthalmology 38(4) (pp 1659-1667), 2018;01.

25. Wang YX, Wei WB, Xu L, Jonas JB. Physical activity and eye diseases. The Beijing Eye Study. Acta Ophthalmol 2019;97:325-31.

26. Lopez Sanchez GF, Mendiola OJ, Torres Cantero AM. Association between Physical Activity and 32 Chronic Conditions among Spanish Adults. International Journal of Environmental Research and Public Health 2019;13596.

27. Pengpid S, Peltzer K. Prevalence and correlates of cataract among a nationally representative population-based sample of older adults in mexico. International Journal on Disability and Human Development 2019;2020.

28. Lopez Sanchez GF, Trott M, Sanchez-Castillo S et al. The association between physical activity and cataract among 1711 older adults residing in Spain. European Geriatric Medicine Conference: 15th International Congress of the European Geriatric Medicine Society, EuGMS 2019;2019.

29. Munch IC, Linneberg A, Larsen M. Precursors of age-related macular degeneration: Associations with physical activity, obesity and plasma lipids in the Inter 99 Eye Study. Acta Ophthalmologica Conference: 40th Nordic Congress of Ophthalmology Helsinki Finland 90(SUPPL 250) (pp 2019;August.

30. Lopez-Sanchez GF, Pardhan S, Trott M et al. The Association Between Physical Activity and Cataracts Among 17,777 People Aged 15-69 Years Residing in Spain. Ophthalmic Epidemiol 2020;27:272-7.

31. Lopez-Sanchez GF, Sanchez-Castillo S, Lopez-Bueno R et al. Comparison of physical activity levels in Spanish people with diabetes with and without cataracts. European journal of public health 30(6) (pp 1201-1205), 2020;11.

32. Kim C-G, Park S, Kim Y. Age-related macular degeneration among the elderly: The 5th National Health and Nutrition Examination Survey, 2010 through 2012. Japan journal of nursing science : JJNS 17(1) (pp e12257), 2020;01.

33. Roustaei GA, Shomali F, Gholinia H, Farzin M, Rasoulinejad SA. Evaluation of impact of life-style-related risk factors in age-related cataract. Acta Medica Iranica 59(2) (pp 91-96), 2021;2021.

34. Katsimpris A, Jurgens C, Ludtke L et al. Association between cardiorespiratory fitness and handgrip strength with age-related macular degeneration: A population-based study. British Journal of Ophthalmology 105(8) (pp 1127-1132), 2021;01.

35. Todkar HS, Katare SS, Wattamwar A, Gumgol S. Prevalence and Risk Factors of Age-Related Macular Degeneration: A Cross-Sectional Study in Urban Populations. International Journal of Tropical Medicine 18(1) (pp 129-133), 2023;2023.

36. Meng Y, Tan Z, Sawut A, Li L, Chen C. Association between Life's Essential 8 and cataract among US adults. Sci Rep 2024;14:13101.

37. Kim Y, Han K, Kim JH. Prevalence and risk factors of undiagnosed age-related macular degeneration: the Korea National Health and Nutrition Examination Survey 2017-2020. Sci Rep 2025;15:10647.

38. Giloyan A, Harutyunyan T, Petrosyan V. Prevalence and risk factors of age-related cataract among adults aged 50 + in Gegharkunik, Armenia. International Ophthalmology 45(1) (no pagination), 2025;114.

39. Klein R, Cruickshanks KJ, Nash SD et al. The prevalence of age-related macular degeneration and associated risk factors. Arch Ophthalmol 2010;128:750-8.

40. Xu Y, Liang A, Zheng X et al. Sex-specific social, lifestyle, and physical health risk factors in cataracts development. Eye (Basingstoke) 38(15) (pp 2939-2946), 2024;01.

41. Qi J, Su K, Zhang K et al. Association of Unhealthy Lifestyles with Cataract Risk, and The Mediating Role of Metabolic Signature: Analysis of the UK Biobank Prospective Cohort. Aging Dis 2025.

42. Wang W, Ren R, Liu Y et al. Life's Essential 8, Genetic Susceptibility, and the Risk of Age-Related Macular Degeneration: A Prospective Cohort Study. Investigative Ophthalmology and Visual Science 66(4) (no pagination), 2025;54.

43. McGuinness MB, Le J, Mitchell P et al. Physical Activity and Age-related Macular Degeneration: A Systematic Literature Review and Meta-analysis. Am J Ophthalmol 2017;180:29-38.

44. Jiang H, Wang LN, Liu Y et al. Physical activity and risk of age-related cataract. Int J Ophthalmol 2020;13:643-9.

45. Mi Y, Zhu Q, Chen Y, Zheng X, Wan M, Li Y. Impact of Physical Activity Frequency, Duration, and Intensity on Senile Cataract Risk: A Mendelian Randomization Study. Transl Vis Sci Technol 2024;13:26.

46. Zhou X, Wu J, Shen Y, He S, Guan H, Shen L. Genetically determined physical activity levels, sedentary behaviours, and their association with the risk of age-related macular degeneration. J Int Med Res 2025;53:3000605251318198.

47. Shen G, Chen Y, Chen J et al. The causal effects of lifestyle, circulating, pigment, and metabolic factors on early age-related macular degeneration: a comprehensive Mendelian randomization study. Journal of Translational Medicine 22(1) (no pagination), 2024;988.

48. Bergman B, Nilsson-Ehle H, Sjostrand J. Ocular changes, risk markers for eye disorders and effects of cataract surgery in elderly people: a study of an urban Swedish population followed from 70 to 97 years of age. Acta Ophthalmol Scand 2004;82:166-74.

49. Meyers KJ, Liu Z, Millen AE et al. Joint Associations of Diet, Lifestyle, and Genes with Age-Related Macular Degeneration. Ophthalmology 2015;122:2286-94.

50. Rim TH, Kim DW, Kim SE, Kim SS. Factors associated with cataract in Korea: A community health survey 2008-2012. Yonsei Medical Journal 56(6) (pp 1663-1670), 2015;01.

51. Torres M, Joslin CE, Kean-Cowdin R, Jiang X, Choudhury F, Varma R. Risk factors for lens opacities in a population-based cohort of adult Chinese Americans: The Chinese American eye study (CHES). Investigative Ophthalmology and Visual Science Conference: 2017;01.

52. Mehta K, Sune P, Belsare A. Study of correlates of age-related macular degeneration in patients attending tertiary hospital. International Journal of Pharmaceutical Research 11(2) (pp 1825-1828), 2019;01.

53. Park JY, Kim JS, Sim HE et al. Prevalence and risk factors of age-related macular degeneration features among pilots. Retina 44(3) (pp 475-486), 2024;01.

54. Kralova JS, Kolar P, Kapounova Z, Vesely P, Brazdova ZD. Lifestyle factors associated with age-related macular degeneration: Case-control study. European Journal of Ophthalmology 34(5) (pp 1548-1554), 2024;01.

55. Yang Z, Zhang J, Zheng Y. Associations Between Life's Essential 8 and Major Ocular Diseases in the American Middle-Aged and Elderly Population. American Journal of Ophthalmology 268(pp 76-85), 2024;01.

56. Song MY, Kim Y, Han K, Kim JH. Prevalence and Risk Factors of Age-Related Macular Degeneration in South Korea: Korea National Health and Nutrition Examination Survey. Ophthalmic Epidemiology 32(1) (pp 34-43), 2025;2025.

57. Klein R, Lee KE, Gangnon RE, Klein BE. Relation of smoking, drinking, and physical activity to changes in vision over a 20-year period: the Beaver Dam Eye Study. Ophthalmology 2014;121:1220-8.

58. Loprinzi PD, Zebardast N, Ramulu PY. Cardiorespiratory fitness and vision loss among young and middle-age U.S. adults. Am J Health Promot 2015;29:226-9.

59. Lee MJ, Wang J, Friedman DS, Boland MV, De Moraes CG, Ramulu PY. Greater Physical Activity Is Associated with Slower Visual Field Loss in Glaucoma. Ophthalmology 2019;126:958-64.

60. McMullan II, Bunting BP, Smith L, Koyanagi A, Tully MA. Is There an Association Between Self-Reported Physical Activity and Self-Rated Vision Over Time? Results From the Irish Longitudinal Study of Ageing. J Aging Phys Act 2020;28:774-81.

61. Gerste RD. Ophthalmology: Physical activity counteracts emergence of cataract. Deutsches Arzteblatt International 112(14) (no pagination), 2015;03.

62. Gerste RD. Age-related macular degeneration: Physically active persons have a lower risk for AMD. Deutsches Arzteblatt International 114(45) (pp A2106), 2017;10.

63. Ruchalla E. Physical activity partially protects against age-related macular degeneration. [German]. Klinische Monatsblatter fur Augenheilkunde 234(2) (pp 153), 2017;01.

64. Subhi Y, Munch IC, Singh A, Sorensen TL. Physical activity benefits patients with age-related macular degeneration. [Danish]. Ugeskrift for laeger 176(37) (no pagination), 2014;08.

65. Subhi Y, Munch IC, Singh A, Sorensen TL. Physical activity benefits patients with age-related macular degeneration. [Danish]. Ugeskrift for laeger 177(34) (pp 1624-1627), 2015;17.

66. Castillo SS, Smith L, Suarez AD, Sanchez GFL. Associations between physical activity and comorbidities in people with COPD residing in Spain: A cross-sectional analysis. International Journal of Environmental Research and Public Health 17(2) (no pagination), 2020;594.

67. Han X, Wu C, Yan X et al. Physical activity and cataract treated surgically in diabetic patients: Findings from the 45 and up study. Investigative Ophthalmology and Visual Science Conference: 2018;01.

68. Wu C, Han X, Yan X, Shang X, Zhang L, He M. Associations between physical activity and cataract treated surgically in patients with diabetes: findings from the 45 and Up Study. Br J Ophthalmol 2019;103:1099-105.

69. Yoon B, Sa H-S, Kim HJ. Incidence and risk factors of age-related macular degeneration in patients with Parkinson's disease: a population-based study. Frontiers in Aging Neuroscience 16(no pagination), 2024;1331786.

70. Klein BEK, Klein R. Lifestyle Exposures and Eye Diseases in Adults. American Journal of Ophthalmology 144(6) (pp 961-969 e1), 2007;December.

71. Gale J, Wells AP, Wilson G. Effects of exercise on ocular physiology and disease. Surv Ophthalmol 2009;54:349-55.

72. Wickremasinghe SS, Chong EWT, Guymer RH. Lifestyle and age-related macular degeneration. Expert Review of Ophthalmology 4(1) (pp 79-102), 2009;2009.

73. Subhi Y, Munch IC, Singh A, Sørensen TL. [Physical activity benefits patients with age-related macular degeneration]. Ugeskr Laeger 2014;176.

74. Subhi Y, Munch IC, Singh A, SÃ¸rensen TL. [Physical activity benefits patients with age-related macular degeneration]. Ugeskr Laeger 2015;177:1624-7.

75. Ong SR, Crowston JG, Loprinzi PD, Ramulu PY. Physical activity, visual impairment, and eye disease. Eye (Lond) 2018;32:1296-303.

76. Li HY, Rong SS, Hong X et al. Exercise and retinal health. Restor Neurol Neurosci 2019;37:571-81.

77. Cui BH, Yan H. Research progress of the effect of exercise on prevention of age-related macular degeneration. [Chinese]. [Zhonghua yan ke za zhi] Chinese journal of ophthalmology 58(12) (pp 1079-1083), 2022;11.

78. Zhang Q, Jiang Y, Deng C, Wang J. Effects and potential mechanisms of exercise and physical activity on eye health and ocular diseases. Front Med (Lausanne) 2024;11:1353624.

79. Brandl C, Heid IM, Helbig H, Holz FG, Finger RP, Mauschitz MM. [Risk factors for age-related macular degeneration : Lessons learned from current data]. Ophthalmologie 2025;122:510-7.

80. Saigal K, Salama JE, Pardo AA, Lopez SE, Gregori NZ. Modifiable Lifestyle Risk Factors and Strategies for Slowing the Progression of Age-Related Macular Degeneration. Vision (Basel) 2025;9.

81. Trott M, Driscoll R, Pardhan S. Associations between diabetic retinopathy and modifiable risk factors: An umbrella review of meta-analyses. Diabet Med 2022;39:e14796.

Supplementary Table 2. Cohort studies of physical activity and cataract

| Author, publication year, country/ region | Study name | Recruitment and follow-up period | Study size, gender, age, number of cases | Exposure, subgroup | Quantity | RR (95% CI) | Adjustment for confounders |
| --- | --- | --- | --- | --- | --- | --- | --- |
| Klein BEK et al, 2003, USA | Beaver Dam Eye Study | 1988-2000, 10.1 years follow-up | 4926 men and women, age 43-86 years: 1535 cataract cases (708 nuclear/617 cortical/ 210 subscapular) | Exercise, nuclear cataract  Exercise, cortical cataract  Exercise, posterior subscapular cataract | >3 times/week  Sedentary  >3 times/week  Sedentary  >3 times/week  Sedentary | 1.00  1.22 (0.97-1.54)  1.00  0.94 (0.75-1.18)  1.00  1.05 (0.73-1.51) | Age, sex |
| Williams PT et al, 2009, USA | National Runners' Health Study | 1993-1993 - NA, 7.6 years follow-up | 40,992 men and women, age  years: 912 cataract cases | Running  Running | <16 km/week  16-32  32-48  48-64  ≥64  <16 km/week  16-32  32-48  48-64  ≥64 | 1.00  0.90 (0.72-1.12)  0.91 (0.72-1.14)  0.74 (0.56-0.96)  0.65 (0.48-0.89)  1.00  0.92 (0.73-1.16)  0.97 (0.77-1.22)  0.80 (0.60-1.07)  0.72 (0.52-1.00) | Age, alcohol, meat, fish, fruit  +BMI |
| Mares JA et al, 2010, USA | Women's Health Initiative | 1994-1998 - 2001-2004, ~6.5 years follow-up | 1808 women, age 50-79 years: 736 nuclear cataract cases | Leisure-time physical activity | <3 METs/d  3-<10  10-<21  ≥21 | 1.00  0.84 (0.61-1.16)  0.87 (0.63-1.20)  0.89 (0.64-1.23) | Age, iris pigmentation, Healthy Eating Index 1995 score, BMI, smoking - pack-years, pulse pressure, energy intake |
| Appleby PN et al, 2011, United Kingdom | European Prospective Investigation into Cancer and Nutrition (EPIC) -Oxford study | 1993-1999 - 2009, 11.4 years follow-up | 27670 nondiabetic men and women, age ≥40 years: 1494 cases | Total physical activity | Inactive  Active | 1.00  0.94 (0.84-1.05) | Age, smoking, ethnicity, self-reported prior high blood pressure, receipt of long-term medical treatment, HRT (women), sex, method of recruitment, region of residence |
| Williams PT, 2013, USA | The National Runners' Health Study II & National Walkers' Health Study | 1998-2001 -2006, 6.4/5.7 years follow-up | 32610 runners and 14917 walkers, age 44.5/54.0 years: 1807 cataract cases | Runners  Running  Walking  Other vigorous  Other moderate  Other light  Runners  Running  Walking  Other vigorous  Other moderate  Other light  Walking and running  Walking and running | vs. walkers  per 1 MET-hours/day  per 1 MET-hours/day  per 1 MET-hours/day  per 1 MET-hours/day  per 1 MET-hours/day  vs. walkers  per 1 MET-hours/day  per 1 MET-hours/day  per 1 MET-hours/day  per 1 MET-hours/day  per 1 MET-hours/day  <1.8 MET-hours/day  1.8-3.6  3.6-5.4  5.4-7.2  7.2-9.0  ≥9.0  <1.8 MET-hours/day  1.8-3.6  3.6-5.4  5.4-7.2  7.2-9.0  ≥9.0 | 0.72 (0.60-0.86)  0.96 (0.94-0.99)  0.92 (0.88-0.96)  0.98 (0.97-1.00)  1.02 (1.00-1.05)  1.02 (0.92-1.02)  0.71 (0.59-0.85)  0.96 (0.94-0.99)  0.92 (0.89-0.96)  0.98 (0.97-1.00)  1.02 (1.00-1.05)  1.02 (0.92-1.02)  1.00  0.84 (0.75-0.94)  0.81 (0.70-0.95)  0.74 (0.61-0.89)  0.66 (0.48-0.89)  0.58 (0.42-0.81)  1.00  0.85 (0.75-0.95)  0.83 (0.71-0.97)  0.76 (0.63-0.92)  0.65 (0.48-0.89)  0.61 (0.44-0.84) | Age, age squared, sex, race, education, smoking, meat, fruit, alcohol  +BMI  +BMI |
| Selin JZ et al, 2015, Sweden | Swedish Mammography Cohort & Cohort of Swedish Men | 1997-1998 - 2011, 12.1 years follow-up | 23852 women and 28807 men, age 45-83 years: 11580 cataract cases | Total physical activity  Walking/bicycling  Exercise  Work/occupational physical activity  Home/housework | 36.7 MET-hours/day  39.8  43.6  47.8  Hardly ever  <20 min/day  20-40  41-60  >60  <1 hour/week  1  2-3  4-5  >5  Mostly sitting  Sitting half of the time  Mostly standing  Mostly walking  Heavy manual labor  <1 hour/day  1-2  3-4  5-6  >6 | 1.00  0.94 (0.89-0.99)  0.91 (0.86-0.96)  0.87 (0.82-0.92)  1.00  0.97 (0.90-1.04)  0.96 (0.90-1.03)  0.95 (0.88-1.02)  0.88 (0.82-0.95)  1.00  0.95 (0.89-1.00)  1.01 (0.96-1.07)  1.00 (0.94-1.08)  0.99 (0.92-1.07)  1.00  0.93 (0.88-0.98)  0.91 (0.83-0.99)  0.92 (0.87-0.97)  0.84 (0.78-0.91)  1.00  1.01 (0.96-1.07)  0.98 (0.92-1.04)  1.02 (0.95-1.11)  1.00 (0.92-1.10) | Age, sex, smoking status, abdominal obesity, history of hypertension, corticosteroid use, education |
| Floud S et al, 2016, United Kingdom | Million Women Study | 1996-2001 - 2008/2011, 11 years follow-up | 1312015 postmenopausal women, age 50-64 years: 89343 cataract surgery cases | Strenuous physical activity | Rarely, never  Some | 1.00  0.90 (0.88-0.91) | Age, region of residence plus deprivation, educational attainment, smoking, BMI, treatment for diabetes, age at menarche, parity, duration of oral contraceptive use, hormone therapy |
| Peng Y et al, 2025, United Kingdom | UK Biobank | 2006-2010 - , 10.8 years follow-up | 426540 men and women, age 37-73 years: 55658 cataract cases | Leisure-time physical activity | <600 MET-minutes/week  600-<1200  ≥1200 | 1.05 (1.02-1.07)  1.05 (1.02-1.07)  1.00 | Age, sex, ethnicity, socioeconomic status, education, BMI, smoking status, alcohol consumption, sun exposure, diabetes, hypertension |

Abbreviations: BMI, body mass index; HRT, hormone replacement therapy; MET, metabolic equivalent task.

Supplementary Table 3. Cohort studies of physical activity and age-related macular degeneration

| Author, publication year, country/ region | Study name | Recruitment and follow-up period | Study size, gender, age, number of cases | Exposure, subgroup | Quantity | RR (95% CI) | Adjustment for confounders |
| --- | --- | --- | --- | --- | --- | --- | --- |
| Knudtson MD et al, 2006, USA | Beaver Dam Eye Study | 1988-1990 -  NA, 15 years follow-up | 3874 men and women, age 43-86 years: 490 early AMD cases  57 geographic atrophy cases  79 exudative AMD cases | Exercise, early AMD  Stair climbing  Walking  Exercise, geographic atrophy  Stair climbing  Walking  Exercise, exudative AMD  Stair climbing  Walking | Sedentary  Active  None  1-3 flights/day  4-6  >6  None  1-4 blocks  5-12  >12  Sedentary  Active  None  1-3 flights/day  4-6  >6  None  1-4 blocks  5-12  >12  Sedentary  Active  None  1-3 fligths/day  4-6  >6  None  1-4 blocks  5-12  >12 | 1.0  0.9 (0.7-1.1)  1.0  -  -  0.9 (0.8-1.0)  1.0  -  -  1.0 (0.9-1.1)  1.0  1.1 (0.5-2.3)  1.0  -  -  1.0 (0.7-1.3)  1.0  -  -  0.9 (0.7-1.2)  1.0  0.3 (0.1-0.7)  1.0  -  -  1.0 (0.8-1.3)  1.0  -  -  0.7 (0.6-0.97) | Age, sex, arthritis, systolic blood pressure, BMI, smoking, education |
| Williams PT et al, 2009, USA | National Runners' Health Study | 1991-1993 - NA, 7.7 years follow-up | 41528 men and women, age ≥18 years: 152 AMD cases | Running  Running | <2 km/day  2-4  4-6  6-8  ≥8  <2 km/day  2-4  4-6  6-8  ≥8 | 1.00  0.81 (0.50-1.32)  0.58 (0.35-0.96)  0.56 (0.30-1.05)  0.47 (0.26-0.84)  1.00  0.79 (0.50-1.29)  0.55 (0.33-0.92)  0.52 (0.27-0.99)  0.41 (0.21-0.78) | Age, age^2^, sex, meat, fish, fruit, alcohol  +BMI |
| Mares JE et al, 2011, USA | Women's Health Initiative | 1994-1998 -  2001-2004, 6 years follow-up | 1313 men and women, age 55-74 years: 202 AMD cases | Leisure-time physical activity | 0-2.2 MET-hours/week  2.4-8  9-13  14-24.5  24.7-119 | 1.00  0.83 (0.51-1.32)  0.71 (0.43-1.17)  0.97 (0.60-1.56)  0.52 (0.30-0.89) | Age, smoking, diabetes, family history of AMD, iris pigment color, cardiovascular disease, hormone therapy use, mHEI score |
| Gopinath B et al, 2014, Australia | Blue Mountains Eye Study | 1992-1994 - 2009, 15 years follow-up | 2037 men and women, age >49 years: 84 late AMD cases | Leisure-time physical activity, <75 years  Leisure-time physical activity, ≥75 years  Leisure-time physical activity, <75 years  Leisure-time physical activity, ≥75 years | ≤198 METs/week  297-1299  >1386  ≤198 METs/week  297-1299  >1386  ≤198 METs/week  297-1299  >1386  ≤198 METs/week  297-1299  >1386 | 1.00  1.11 (0.45-2.75)  0.25 (0.05-1.17)  1.00  1.31 (0.66-2.62)  0.99 (0.47-2.06)  1.00  1.03 (0.40-2.65)  0.26 (0.06-1.28)  1.00  1.43 (0.71-2.90)  1.04 (0.48-2.24) | Age, sex, smoking, fish consumption  + white cell count, BMI |
| McGuinnes MB et al, 2016, Australia | Melbourne Collaborative Cohort Study | 1990-1994 - 2003-2007, 11.3 years follow-up | 20816 men and women, age 40-69 years: 4244 early AMD cases  2661 intermediate AMD cases  122 late AMD cases | Total recreational physical activity, early AMD  Vigorous exercise, men  Vigorous exercise, women  Total recreational physical activity, intermediate AMD  Vigorous exercise, men  Vigorous exercise, women  Total recreational physical activity, late AMD  Vigorous exercise, men  Vigorous exercise, women | 0 MET-minutes/week  >0-<500  500-<1000  1000-2550  None  1-2 times/week  ≥3 times/week  None  1-2 times/week  ≥3 times/week  0 MET-minutes/week  >0-<500  500-<1000  1000-2550  None  1-2 times/week  ≥3 times/week  None  1-2 times/week  ≥3 times/week  0 MET-minutes/week  >0-<500  500-<1000  1000-2550  None  1-2 times/week  ≥3 times/week  None  1-2 times/week  ≥3 times/week | 1.00  1.04 (0.93-1.17)  1.05 (0.95-1.16)  1.07 (0.95-1.19)  1.00  0.97 (0.81-1.15)  0.95 (0.81-1.12)  1.00  0.98 (0.87-1.11)  0.97 (0.84-1.12)  1.00  0.96 (0.84-1.10)  0.97 (0.86-1.09)  0.94 (0.82-1.08)  1.00  0.93 (0.75-1.14)  1.01 (0.84-1.23)  1.00  0.98 (0.84-1.14)  0.78 (0.64-0.96)  1.00  1.02 (0.56-1.85)  1.09 (0.65-1.84)  1.20 (0.66-2.20)  1.00  1.12 (0.44-2.87)  0.61 (0.19-1.99)  1.00  0.53 (0.21-1.33)  0.76 (0.27-2.11) | Age, sex, smoking status, region of descent, Mediterranean diet score |
| Gopinath B et al, 2017, Australia | Blue Mountains Eye Study | 1992-1994 - 2007-2009, 15 years follow-up | 1903 men and women, age >49 years: 354 early AMD cases  118 late AMD cases | Leisure-time physical activity, early AMD  Leisure-time physical activity, late AMD | <3 vs. ≥3 episodes/week  <3 vs. ≥3 episodes/week | 0.96 (0.75-1.24)  1.01 (0.64-1.61) | Age, sex, white cell count, fish consumption |
| Rim TH et al, 2018, Korea | South Korea National Health Insurance Service database | 2002-2003 - 2009-2013, 4.2 years follow-up | 211960 men and women, age 45-79 years: 448 neovascular AMD cases | Vigorous physical activity, all  Vigorous physical activity, men  Vigorous physical activity, women  Vigorous physical activity, all  Vigorous physical activity, men  Vigorous physical activity, women | No  Yes  No  Yes  No  Yes  No  1-4 times/week  ≥5 times/week  No  1-4 times/week  ≥5 times/week  No  1-4 times/week  ≥5 times/week | 1.00  1.23 (1.02-1.49)  1.00  1.36 (1.09-1.69)  1.00  0.90 (0.62-1.32)  1.00  1.17 (0.95-1.44)  1.38 (1.07-1.79)  1.00  1.28 (1.01-1.63)  1.54 (1.15-2.06)  1.00  0.86 (0.59-1.33)  1.00 (0.59-1.67) | Age, BMI, systolic blood pressure, diastolic blood pressure, fasting glucose, total cholesterol, hemoglobin, aspartate aminotransferase, alanine transaminase, Charlson Comorbidity Index, Annual Medical care visits, annual ophthalmologist visits, sex, income, residence, alcohol use, smoking, hypertension, hyperlipidemia, acute myocardial infarction, heart failure, peripheral vascular disease, cerebrovascular disease, chronic pulmonary disease, liver diseases, uncomplicated diabetes, complicated diabetes, cancer, prescription drug use - anti-diabetes medication, anti-hypertensives, statins, aspirin |
| Mauschitz MM et al, 2021, Germany | Gutenberg Health Study | 2007-2017 - NA, 5.0 years follow-up | 3978 men and women, age 50-74 years: 121 early AMD cases | Leisure-time physical activity | No/low/moderate  High | 1.74 (1.21-2.51)  1.00 | Sex, smoking status |
| Mauschitz MM et al, 2021, Germany | KORA study | 1999-2014 - NA, 13.5 years follow-up | 340 men and women, age 54-75 years: 122 early AMD cases | Leisure-time physical activity | No/low/moderate  High | 1.42 (0.89-2.26)  1.00 | Sex, smoking status |
| Mauschitz MM et al, 2021, Netherlands | Rotterdam Studies I-III | 1997-2009 - NA, 10.8 years follow-up | 6192 men and women, age 50-93 years: 662 early AMD cases | Leisure-time physical activity | No/low/moderate  High | 1.00 (0.85-1.17)  1.00 | Sex, smoking status |
| Mauschitz MM et al, 2021, France | POLA Study | 1995-2000 - NA, 3.0 years follow-up | 1368 men and women, age 60-92 years: 115 early AMD cases | Leisure-time physical activity | No/low/moderate  High | 1.05 (0.71-1.55) 1.00 | Sex, smoking status |
| Mauschitz MM et al, 2021, Germany | AugUR Study | 2013-2015 - NA, 3.2 years follow-up | 620 men and women, age 70-95 years: 61 early AMD cases | Leisure-time physical activity | No/low/moderate  High | 1.05 (0.56-1.98)  1.00 | Sex, smoking status |
| Mauschitz MM et al, 2021, France | Alienor Study | 2009-2015 - NA, 4.3 years follow-up | 311 men and women, age 76-96 years: 29 early AMD cases | Leisure-time physical activity | No/low/moderate  High | 1.60 (0.69-3.69) | Sex, smoking status |
| Ludtke L et al, 2024, Germany | Study of Health in Pomerania (SHIP)-TREND-1 | 2008-2012 - 2016-2019, ~7.5 years follow-up | 1418 men and women, age 20-79 years: 148 AMD cases | Physical activity, all  Physical activity, men  Physical activity, women | High vs. low  High vs. low  High vs. low | 1.02 (0.67-1.55)  0.67 (0.38-1.20)  1.55 (0.83-2.88) | Age |
| Peng Y et al, 2025, United Kingdom | UK Biobank | 2006-2010 - 2020-2021, 10.9 years follow-up | 271274 men and women, age 37-73 years: 7468 AMD cases | Leisure-time physical activity | Non-ideal  Ideal (>600 MET-minutes/week) | 1.00  0.93 (0.88-0.98) | Age, sex, ethnicity, Townsend deprivation index, education, household income, drinking status, diet, tobacco smoking, sleep health, BMI, blood lipids, blood glucose, blood pressure |

Abbreviations: AMD, age-related macular degeneration; BMI, body mass index; mHEI, modified healthy eating index; MET, metabolic equivalent task; NA, not available.

Supplementary Table 4. Study quality assessment using modified Newcastle-Ottawa scale for studies on physical activity and cataract

| Author, publication year, country/ region | Selection | | | Comparability | Outcome assessment | | | Total |
| --- | --- | --- | --- | --- | --- | --- | --- | --- |
|  | Selection of non-exposed cohort | Exposure ascertainment | Demonstration of outcome not present at start | 0.25 points for each adjustment | Outcome assessment | Long enough follow-up (≥3 years) | Adequacy of follow-up (<10% lost) |  |
| Klein BEK et al, 2003, USA | 1 | 0 | 0 | 0.5 | 1 | 1 | 0 | 3.5 |
| Williams PT, 2009, USA | 1 | 0 | 0 | 1.25 | 0 | 1 | 0 | 3.25 |
| Mares JA et al, 2010, USA | 1 | 0 | 0 | 1.75 | 1 | 1 | 0 | 4.75 |
| Appleby PN et al, 2011, United Kingdom | 1 | 0 | 1 | 2 | 1 | 1 | 1 | 7 |
| Williams PT, 2013, USA | 1 | 0 | 1 | 2 | 0 | 1 | 0 | 5 |
| Selin JZ et al, 2015, Sweden | 1 | 1 | 1 | 1.75 | 1 | 1 | 0 | 6.75 |
| Floud S et al, 2016, United Kingdom | 1 | 0 | 1 | 2 | 1 | 1 | 1 | 7 |
| Peng Y et al, 2025, United Kingdom | 1 | 1 | 1 | 2 | 1 | 1 | 1 | 8 |

Supplementary Table 5. Modified Newcastle-Ottawa Scale score for studies on physical activity and age-related macular degeneration

| Author, publication year, country/ region | Selection | | | Comparability | Outcome assessment | | | Total |
| --- | --- | --- | --- | --- | --- | --- | --- | --- |
|  | Selection of non-exposed cohort | Exposure ascertainment | Demonstration of outcome not present at start | 0.25 points for each adjustment | Outcome assessment | Long enough follow-up (≥3 years) | Adequacy of follow-up (<10% lost) |  |
| Knudtson MD et al, 2006, USA | 1 | 0 | 0 | 1.75 | 1 | 1 | 0 | 4.75 |
| Williams PT et al, 2009, USA | 1 | 0 | 1 | 1.75 | 0 | 1 | 0 | 4.75 |
| Mares JE et al, 2011, USA | 1 | 0 | 0 | 2 | 1 | 1 | 0 | 5 |
| McGuinnes MB et al, 2016, Australia | 1 | 0 | 0 | 1.25 | 1 | 1 | 0 | 4.25 |
| Gopinath B et al, 2017, Australia | 1 | 0 | 0 | 1 | 1 | 1 | 0 | 4 |
| Rim TH et al, 2018, Korea | 1 | 1 | 1 | 2 | 1 | 1 | 0 | 7 |
| Mauschitz MM et al, 2021, Germany | 1 | 0 | 1 | 0.5 | 1 | 1 | 0 | 4.5 |
| Mauschitz MM et al, 2021, Germany | 1 | 0 | 1 | 0.5 | 1 | 1 | 0 | 4.5 |
| Mauschitz MM et al, 2021, Netherlands | 1 | 0 | 1 | 0.5 | 1 | 1 | 0 | 4.5 |
| Mauschitz MM et al, 2021, France | 1 | 0 | 1 | 0.5 | 1 | 1 | 0 | 4.5 |
| Mauschitz MM et al, 2021, Germany | 1 | 0 | 1 | 0.5 | 1 | 1 | 0 | 4.5 |
| Mauschitz MM et al, 2021, France | 1 | 0 | 1 | 0.5 | 1 | 1 | 0 | 4.5 |
| Ludtke L et al, 2024, Germany | 1 | 0 | 0 | 0.25 | 1 | 1 | 0 | 3.25 |
| Peng Y et al, 2025, United Kingdom | 1 | 1 | 1 | 2 | 1 | 1 | 1 | 8 |

Supplementary Table 6. World Cancer Research Fund grading criteria

| Grading | Criteria |
| --- | --- |
| Convincing | A convincing relationship should be robust enough to be highly unlikely to be modified in the foreseeable future as new evidence accumulates. All of the following are generally required:  - Evidence from more than one study type  - Evidence from at least two independent cohort studies  - No substantial unexplained heterogeneity within or between study types or in different populations relating to the presence or absence of an association, or direction of effect  - Good quality studies to exclude with confidence the possibility that the observed association results from random or systematic error, including confounding, measurement error, and selection bias  - Presence of a plausible biological gradient in the association. Such a gradient need not be linear or even in the same direction across different levels of exposure, so long as this can be explained plausibly  - Strong and plausible experimental evidence, either from human studies or relevant animal models, that typical human exposures can lead to relevant outcomes |
| Probable | All of the following are generally required:  - Evidence from at least two independent cohort studies, or at least five case-control studies  - No substantial unexplained heterogeneity within or between study types or in different populations relating to the presence or absence of an association, or direction of effect  - Good quality studies to exclude with confidence the possibility that the observed association results from random or systematic error, including confounding, measurement error, and selection bias  - Evidence for biological plausibility |
| Limited - suggestive | All of the following are generally required:  - Evidence from at least two independent cohort studies, or at least five case-control studies  - The direction of effect is generally consistent though some unexplained heterogeneity may be present  - Evidence for biological plausibility |
| Limited - no conclusion | Evidence is so limited that no firm conclusion can be made, but this does not mean that there is evidence of no relationship. The evidence might be graded "limited - no conclusion" for several reasons:  - limited number of studies  - inconsistency of direction of effect  - poor quality of studies (e.g. lack of adjustment for known confounders)  - or any combination of these factors |
| Substantial effect on risk unlikely | All of the following are generally required:  - Evidence from more than one study type  - Evidence from at least two independent cohort studies  - Summary estimate of effect close to 1.0 for comparison of high versus low exposure categories  - No substantial unexplained heterogeneity within or between study types or in different populations  - Good quality studies to exclude with confidence the possibility that the absence of association results from random or systematic error, including inadequate power, imprecision or error in exposure measurement, inadequate range of exposure, confounding, and selection bias  - Absence of a demonstrable biological gradient (dose response)  - Absence of strong and plausible experimental evidence, either from human studies or relevant animal models, that typical human exposures lead to relevant outcomes |

Specific upgrading factors:

1) Presence of a plausible biological gradient (dose response) in the association. Such a gradient need not be linear or even in the same direction across the different levels of exposure, so long as this can be explained plausibly.

2) A particularly large summary effect size (an odds ratio or relative risk of 2.0 or more, depending on the unit of exposure) after appropriate control for confounders.

3) Evidence from randomised trials in humans.

4) Evidence from appropriately controlled experiments demonstrating one or more plausible and specific mechanisms actually operating in humans.

5) Robust and reproducible evidence from experimental studies in appropriate animal models showing that typical human exposures can lead to relevant health outcomes.

Supplementary Table 7. Table of relative risks (95% CIs) from nonlinear dose-response analysis in relation to cataract and age-related macular degeneration

| MET-hours/week | Cataract (n=3) | Age--related macular degeneration (n=2) |
| --- | --- | --- |
|  | RR (95% CI) | RR (95% CI) |
| 0 | 1.00 (1.45 MET-hrs/week) | 1.00 |
| 5 | 0.99 (0.95-1.02) | 1.01 (0.97-1.06) |
| 10 | 0.97 (0.91-1.05) | 1.02 (0.94-1.10) |
| 15 | 0.96 (0.86-1.06) | 1.01 (0.92-1.11) |
| 20 | 0.94 (0.83-1.06) | 0.98 (0.87-1.11) |
| 25 | 0.91 (0.79-1.05) | 0.95 (0.80-1.13) |
| 30 | 0.89 (0.77-1.02) | 0.91 (0.72-1.16) |
| 35 | 0.85 (0.74-0.99) |  |
| 40 | 0.82 (0.71-0.95) |  |
| 45 | 0.79 (0.69-0.90) |  |
| 50 | 0.75 (0.66-0.87) |  |
| 55 | 0.72 (0.62-0.83) |  |
| 60 | 0.69 (0.59-0.81) |  |
| 65 | 0.66 (0.55-0.79) |  |
| 70 | 0.63 (0.52-0.77) |  |
| p_nonlinearity_ | 0.32 | 0.34 |

Supplementary Table 8. Subgroup analyses of physical activity and cataract and age-related macular degeneration

|  | | Cataract | | | | | | Age-related macular degeneration | | | | | |
| --- | --- | --- | --- | --- | --- | --- | --- | --- | --- | --- | --- | --- | --- |
|  | | n | RR (95% CI) | | I^2^ | P_h_^1^ | P_h_^2^ | n | RR (95% CI) | I^2^ | P_h_^1^ | P_h_^2^ | |
| All studies | | 8 | 0.90 (0.86-0.94) | | 73.7 | <0.001 |  | 14 | 0.92 (0.84-1.01) | 54.7 | 0.009 |  | |
| Duration of follow-up | |  |  | |  |  |  |  |  |  |  |  | |
| <10 years follow-up | | 3 | 0.69 (0.54-0.89) | | 44.1 | 0.17 | 0.04 | 8 | 0.78 (0.58-1.05) | 73.0 | 0.001 | 0.34 | |
| ≥10 years follow-up | | 5 | 0.91 (0.88-0.95) | | 72.9 | 0.005 |  | 6 | 0.96 (0.91-1.01) | 17.9 | 0.30 |  |  |
| Gender | |  |  | |  |  |  |  |  |  |  |  | |
| Men | | 0 |  | |  |  | 0.90 | 2 | 0.97 (0.54-1.73) | 74.1 | 0.05 | 0.12 | |
| Women | | 2 | 0.90 (0.89-0.92) | | 0 | 0.95 |  | 2 | 1.25 (1.05-1.50) | 0 | 0.49 |  |  |
| Men and women | | 6 | 0.88 (0.82-0.96) | | 76.0 | 0.001 |  | 12 | 0.89 (0.81-0.98) | 48.0 | 0.04 |  |  |
| Geographic location | |  |  | |  |  |  |  |  |  |  |  | |
| Europe | | 4 | 0.91 (0.88-0.95) | | 79.4 | 0.002 | 0.30 | 8 | 0.90 (0.80-1.01) | 30.5 | 0.19 | 0.54 | |
| America | | 4 | 0.77 (0.60-0.98) | | 69.6 | 0.02 |  | 3 | 0.64 (0.41-0.99) | 66.7 | 0.05 |  |  |
| Australia | | 0 |  | |  |  |  | 2 | 1.01 (0.94-1.09) | 0 | 0.87 |  |  |
| Asia | | 0 |  | |  |  |  | 1 | 1.23 (1.02-1.49) |  |  |  |  |
| Number of cases | |  |  | |  |  |  |  |  |  |  |  | |
| Cases <500 | | 0 |  | |  |  | 0.32 | 10 | 0.81 (0.65-1.01) | 68.0 | 0.001 | 0.38 | |
| Cases 500-<1000 | | 2 | 0.76 (0.56-1.03) | | 45.0 | 0.18 |  | 2 | 0.95 (0.83-1.08) | 3.5 | 0.31 |  |  |
| Cases ≥1000 | | 6 | 0.91 (0.87-0.95) | | 77.6 | <0.001 |  | 2 | 0.96 (0.89-1.04) | 65.3 | 0.09 |  |  |
| AMD outcome type | |  |  | |  |  |  |  |  |  |  |  | |
| Total AMD | | - |  | |  |  |  | 4 | 0.75 (0.54-1.05) | 68.9 | 0.02 | 0.16 | |
| Early AMD | | - |  | |  |  |  | 9 | 0.93 (0.82-1.05) | 45.6 | 0.07 |  |  |
| Intermediate AMD | | - |  | |  |  |  | 1 | 0.94 (0.82-1.08) |  |  |  |  |
| Late AMD | | - |  | |  |  |  | 2 | 1.06 (0.74-1.53) | 0 | 0.62 |  |  |
| Dry AMD | | - |  | |  |  |  | 1 | 1.10 (0.50-2.30) |  |  |  |  |
| Wet AMD | | - |  | |  |  |  | 2 | 0.66 (0.17-2.61) | 87.2 | 0.005 |  |  |
| Study quality | |  |  | |  |  |  |  |  |  |  |  | |
| 0-3 stars | | 0 |  | |  |  | 0.30 | 0 |  |  |  | 0.20 | |
| >3-6 | | 4 | 0.77 (0.60-0.98) | | 69.6 | 0.02 |  | 12 | 0.87 (0.76-0.98) | 54.7 | 0.01 |  |  |
| >6-8 | | 4 | 0.91 (0.88-0.95) | | 79.4 | 0.002 |  | 2 | 1.05 (0.80-1.38) | 87.1 | 0.005 |  |  |
| Adjustment for confounders | | | | | | | | | | | | | |
| Age | Yes | 8 | | 0.90 (0.86-0.94) | 73.7 | <0.001 | NC | 8 | 0.96 (0.86-1.07) | 67.3 | 0.003 | 0.38 |  |
|  | No | 0 | |  |  |  |  | 6 | 0.82 (0.65-1.02) | 47.2 | 0.09 |  |  |
| Education | Yes | 4 | | 0.90 (0.85-0.95) | 86.3 | <0.001 | 0.93 | 2 | 0.93 (0.88-0.98) | 0 | 0.55 | 0.89 |  |
|  | No | 4 | | 0.89 (0.79-1.01) | 37.3 | 0.19 |  | 12 | 0.90 (0.78-1.03) | 62.2 | 0.002 |  |  |
| Socioeconomic status | Yes | 2 | | 0.92 (0.88-0.97) | 91.4 | 0.001 | 0.40 | 1 | 0.93 (0.88-0.98) |  |  | 0.81 |  |
|  | No | 6 | | 0.85 (0.77-0.95) | 58.7 | 0.03 |  | 13 | 0.90 (0.80-1.02) | 60.5 | 0.002 |  |  |
| Smoking | Yes | 5 | | 0.90 (0.85-0.95) | 81.7 | <0.001 | 0.92 | 11 | 0.93 (0.84-1.02) | 61.4 | 0.004 | 0.96 |  |
|  | No | 3 | | 0.88 (0.76-1.03) | 58.0 | 0.09 |  | 3 | 0.85 (0.57-1.27) | 67.6 | 0.05 |  |  |
| Alcohol | Yes | 3 | | 0.73 (0.51-1.04) | 86.0 | 0.001 | 0.30 | 3 | 0.92 (0.69-1.25) | 84.9 | 0.001 | 0.63 |  |
|  | No | 5 | | 0.90 (0.88-0.91) | 0 | 0.69 |  | 11 | 0.90 (0.80-1.01) | 46.2 | 0.05 |  |  |
| Fruit and/or vegetables | Yes | 2 | | 0.61 (0.49-0.77) | 0 | 0.62 | 0.02 | 1 | 0.47 (0.26-0.84) |  |  | 0.08 |  |
|  | No | 6 | | 0.91 (0.88-0.95) | 66.2 | 0.01 |  | 13 | 0.94 (0.86-1.03) | 54.7 | 0.009 |  |  |
| Adjustment for potential intermediate factors | | | | | | | | | | | | |  |
| Body mass index | Yes | 4 | | 0.91 (0.87-0.95) | 79.1 | 0.002 | 0.58 | 3 | 0.99 (0.83-1.18) | 76.1 | 0.02 | 0.28 |  |
|  | No | 4 | | 0.80 (0.66-0.98) | 74.9 | 0.008 |  | 11 | 0.86 (0.74-0.99) | 57.3 | 0.009 |  |  |
| Diabetes | Yes | 2 | | 0.92 (0.88-0.97) | 91.4 | 0.001 | 0.40 | 3 | 0.94 (0.70-1.25) | 83.9 | 0.002 | 0.60 |  |
|  | No | 6 | | 0.85 (0.77-0.95) | 58.7 | 0.03 |  | 11 | 0.90 (0.80-1.01) | 48.4 | 0.04 |  |  |
| Hypertension/blood pressure | Yes | 3 | | 0.92 (0.86-0.98) | 73.1 | 0.02 | 0.44 | 3 | 0.99 (0.83-1.18) | 76.1 | 0.02 | 0.28 | |
|  | No | 5 | | 0.83 (0.72-0.95) | 65.0 | 0.02 |  | 11 | 0.86 (0.74-0.99) | 57.3 | 0.009 |  |  |

N denotes the number of risk estimates.

^1^ P for heterogeneity within each subgroup,

^2^ P for heterogeneity between subgroups with meta-regression analysis,

NC = not calculable

AMD; age-related macular degeneration

Supplementary Table 9. Evidence grading for physical activity and cataract and age-related macular degeneration

|  | Reduced risk | Increased risk |
| --- | --- | --- |
| Convincing | - | - |
| Probable | Cataract | - |
| Limited-suggestive | - | - |
| Limited - no conclusion | Age-related macular degeneration | |

Supplementary Table 10. Justification for evidence grading of physical activity and cataract and age-related macular degeneration

| Requirements for grading of convincing | Cataract | Age-related macular degeneration |
| --- | --- | --- |
| Statistically significant and robust association | Statistically significant 10% reduction in risk | No statistically significant association |
| Evidence from at least two independent cohort studies | 10 cohort studies included | 14 cohort studies included |
| No substantial unexplained heterogeneity within or between study types or in different populations relating to the presence or absence of an association, or direction of effect | High heterogeneity (I^2^=74%), but this is due to differences in the size of the association, not different direction of the associations. I^2^ is reduced to 0% when excluding two outlying studies. | Moderate heterogeneity (I^2^=60%) |
| Good quality studies to exclude with confidence the possibility that the observed association results from random or systematic error, including confounding, measurement error, and selection bias | No indication of publication bias with Egger's test or by inspection of the funnel plot.  Studies have adjusted for most important confounders and results persisted across most subgroup analyses.  All studies have used a baseline assessment of physical activity, which likely may have attenuated the observed associations.  Most studies (5/8) stated that they excluded prevalent cataract cases at baseline. Exposed and non-exposed participants were selected from the same populations. | No indication of publication bias.  Most studies have adjusted for important confounders. Little heterogeneity in subgroup analyses by study characteristics and adjustments and there is no clear association across most subgroup analyses.  All studies have used a baseline assessment of physical activity, which likely may have attenuated the observed associations. |
| Presence of a plausible biological gradient in the association. Such a gradient need not be linear or even in the same direction across different levels of exposure, so long as this can be explained plausibly | Evidence of a strong inverse dose-response relationship in the linear dose-response analysis and nonlinear dose-response analysis, however, only three studies could be included in the dose-response analysis. | No significant association in linear or nonlinear dose-response analyses. |
| Strong and plausible experimental evidence, either from human studies or relevant animal models, that typical human exposures can lead to relevant outcomes | - Reduced insulin resistance/diabetes risk and reduced adiposity  - Reduced inflammation  - Improvements in ocular circulation | - Reduced insulin resistance/diabetes risk and reduced adiposity  - Reduced inflammation  - Improvements in ocular circulation |
| Final grading and justification for overall assessment. | Probable evidence that physical activity reduce the risk of cataract.  Justification: Primarily based on highly significant main analysis - although heterogeneity is high it is not unexplained. Biologically plausible mechanisms exist. | There is limited evidence and no conclusion can be made with regard to the association between physical activity and age-related macular degeneration.  Justification: Primarily based on no clear association observed in the main analysis. Although biologically plausible mechanisms exist, this is not sufficient for a stronger grading. |

Supplementary figure 1. Influence analysis of physical activity and cataract

------------------------------------------------------------------------------

Study omitted | e^coef. [95% Conf. Interval]

-------------------+----------------------------------------------------------

Peng, 2025, UKB | 0.88141072 0.83212024 0.93362087

Floud, 2016, MWS | 0.88563865 0.8204909 0.95595926

Selin, 2015, SMC & COSM| 0.90763551 0.86044848 0.95741034

Williams, 2013, NRHSII & NWHS| 0.90980721 0.87392437 0.94716334

Appleby, 2011, EPIC-Oxford| 0.89502418 0.85109317 0.94122279

Mares, 2010, WHI | 0.90036273 0.85862356 0.94413096

Williams, 2009, NRHS| 0.90735626 0.86863893 0.94779932

Klein, 2003, BDES | 0.89687884 0.85390717 0.94201291

-------------------+----------------------------------------------------------

Combined | 0.90063745 0.86006114 0.94312809

------------------------------------------------------------------------------

Supplementary figure 2. Funnel plot of physical activity and cataract

Supplementary figure 3. Influence analysis of physical activity and age-related macular degeneration

------------------------------------------------------------------------------

Study omitted | e^coef. [95% Conf. Interval]

-------------------+----------------------------------------------------------

Peng, 2025, UK Biobank| 0.89912021 .79504865 1.0168147

Ludtke, 2024, SHIP-TREND-I| 0.91800529 .83362532 1.0109262

Mauschitz, 2022, Alienor| 0.92750931 .8447485 1.0183783

Mauschitz, 2022, AugUR| 0.92143869 .83767837 1.0135742

Mauschitz, 2022, GHS| 0.95381892 .87670475 1.037716

Mauschitz, 2022, KORA| 0.93281698 .84943223 1.0243872

Mauschitz, 2022, POLA| 0.92037565 .8354246 1.013965

Mauschitz, 2022, RSI-III| 0.90897334 .81967604 1.0079989

Rim, 2018, SKNHISD| 0.90167183 .8243475 0.98624927

Gopinath, 2017, BMES| 0.91039032 .82362062 1.0063014

McGuinnes, 2016, MCCS| 0.89369291 .79597449 1.0034078

Mares, 2011, WHI | 0.94172448 .86217791 1.0286102

Williams, 2009, NRHS| 0.94305283 .86473119 1.0284684

Knudtson, 2006, BDES| 0.92685628 .83863688 1.0243559

-------------------+----------------------------------------------------------

Combined | 0.92361528 .84183941 1.0133348

------------------------------------------------------------------------------

Supplementary figure 4. Funnel plot of physical activity and age-related macular degeneration
